# Supplementary material for: How does domestic migration pose a challenge in achieving equitable social health insurance benefits in China? A national cross-sectional study
Source: BMJ Open. 2022 Aug 23;12(8):e060551. doi: 10.1136/bmjopen-2021-060551 (PMC9403113; doi:10.1136/bmjopen-2021-060551)
Supplement: Supplementary data [file bmjopen-2021-060551supp001.pdf]

Appendix Table 1 Characteristics of the study sample by migration scope

	Across counties within a city	Across cities within a province	Across provinces	F/χ2	P value
Age (Years) *	38.41(±10.35)	37.99(±9.62)	37.01 (±9.47)	2.248	0.106
Female	137(46.28%)	154(46.0%)	241(45.13%)	0.12	0.942
Married	262(88.51%)	298(88.96%)	480(89.89%)	0.424	0.809
Education attainment				6.979	0.323
Primary school and below	72(24.32%)	78(23.28%)	129(24.16%)		
Junior high school	137(46.28%)	154(45.97%)	263(49.25%)		
Senior high school	64(21.62%)	62(18.51%)	86(16.10%)		
College and above	23(7.77%)	41(12.24%)	56(10.49%)		
Monthly household income per capita (Yuan)*	1830.88(±1272.07)	1968.94(±1589.62)	2672.67(±2600.04)	20.506	0.000
Having any job	226(76.35%)	264(78.81%)	437(81.84%)	3.692	0.158
Rural Hukou	268(90.54%)	293(87.46%)	462(86.52%)	2.934	0.231
Living in an urban area	237(80.07%)	241(71.94%)	330(61.80%)	31.386	0.000
Reasons for migration				8.13	0.087
Seeking jobs	242(81.76%)	273(81.49%)	466(87.27%)		
Family members following migrants	44(14.86%)	51(15.22%)	51(9.55%)		
Other reasons	10(3.38%)	11(3.28%)	17(3.18%)		
Migration duration (Years)				10.475	0.106
0~1	43(14.53%)	32(9.55%)	72(13.48%)		
1~5	135(45.61%)	135(40.30%)	214(40.07)		
5~10	65(21.96%)	80(23.88%)	127(23.78%)		
10 +	53(17.91%)	88(26.27%)	121(22.66%)		
Social health insurance programs				8.376	0.079
NCMS	216(72.97%)	214(63.88%)	347(64.98%)		
URBMI	28(9.50%)	39(11.64%)	53(9.93%)		
UEBMI	52(17.57%)	82(24.48%)	134(25.09%)		

Note: * mean (± Standard Deviation).
