# Supplementary material for: How does domestic migration pose a challenge in achieving equitable social health insurance benefits in China? A national cross-sectional study
Source: BMJ Open. 2022 Aug 23;12(8):e060551. doi: 10.1136/bmjopen-2021-060551 (PMC9403113; doi:10.1136/bmjopen-2021-060551)
Supplement: Author's manuscript [file bmjopen-2021-060551.draft_revisions.pdf]

BMJ Open

BMJ Open is committed to open peer review. As part of this commitment we make the peer review history of every article we publish publicly available.

When an article is published we post the peer reviewers' comments and the authors' responses online. We also post the versions of the paper that were used during peer review. These are the versions that the peer review comments apply to.

The versions of the paper that follow are the versions that were submitted during the peer review process. They are not the versions of record or the final published versions. They should not be cited or distributed as the published version of this manuscript.

BMJ Open is an open access journal and the full, final, typeset and author-corrected version of record of the manuscript is available on our site with no access controls, subscription charges or pay-per-view fees (http://bmjopen.bmj.com).

If you have any questions on BMJ Open's open peer review process please email info.bmjopen@bmj.com

BMJ Open

How does domestic migration pose challenge in achieving equitable social health insurance benefits in China: a National Cross-Sectional Study

Journal:	*BMJ Open*
Manuscript ID	bmjopen-2021-060551
Article Type:	Original research
Date Submitted by the Author:	03-Jan-2022

Complete List of Authors:	Hou, Zhiyuan; Fudan University<br>Wang, Haiqin; The International Peace Maternity & Child Health Hospital of China Welfare Institute, ; Fudan University School of Public Health,<br>Liang, Di; University of California San Diego, Department of Family Medicine and Public Health<br>Zhang, Donglan; University of Georgia, Department of Health Policy and Management, College of Public Health

Keywords:	Health policy < HEALTH SERVICES ADMINISTRATION & MANAGEMENT, HEALTH SERVICES ADMINISTRATION & MANAGEMENT, Health economics < HEALTH SERVICES ADMINISTRATION & MANAGEMENT

SCHOLARONE™
Manuscripts

I, the Submitting Author has the right to grant and does grant on behalf of all authors of the Work (as defined in the below author licence), an exclusive licence and/or a non-exclusive licence for contributions from authors who are: i) UK Crown employees; ii) where BMJ has agreed a CC-BY licence shall apply, and/or iii) in accordance with the terms applicable for US Federal Government officers or employees acting as part of their official duties; on a worldwide, perpetual, irrevocable, royalty-free basis to BMJ Publishing Group Ltd ("BMJ") its licensees and where the relevant Journal is co-owned by BMJ to the co-owners of the Journal, to publish the Work in this journal and any other BMJ products and to exploit all rights, as set out in our licence.

The Submitting Author accepts and understands that any supply made under these terms is made by BMJ to the Submitting Author unless you are acting as an employee on behalf of your employer or a postgraduate student of an affiliated institution which is paying any applicable article publishing charge ("APC") for Open Access articles. Where the Submitting Author wishes to make the Work available on an Open Access basis (and intends to pay the relevant APC), the terms of reuse of such Open Access shall be governed by a Creative Commons licence – details of these licences and which Creative Commons licence will apply to this Work are set out in our licence referred to above.

Other than as permitted in any relevant BMJ Author's Self Archiving Policies, I confirm this Work has not been accepted for publication elsewhere, is not being considered for publication elsewhere and does not duplicate material already published. I confirm all authors consent to publication of this Work and authorise the granting of this licence.

For peer review only

How does domestic migration pose challenge in achieving equitable social health insurance benefits in China: a National Cross-Sectional Study

Abstract

Objectives In China, the mass internal migration since 1980s has posed challenges for the social health insurance to ensure equitable benefits for migrant population. This study evaluated the benefit distribution of social health insurance among internal migrants in China.

Methods Data used for this analysis were from the 2014 China National Internal Migrant Dynamic Monitoring Survey. Descriptive analyses were used to described the general characteristics of our study sample. By applying a two-part model: Chi-square test and one-way variance analysis were used to compare the differences of the probability of receiving reimbursement, the amount and ratio of reimbursement received according to the geographic scope of migration.

Results We found that the broader the geographic scope of migration, the lower the probability of receiving reimbursements from the social health insurance and the reimbursement ratio; but among those who received reimbursements, the broader the geographic scope of migration, the larger amounts they were reimbursed for health care use

Conclusions We attributed this unequal benefit distribution to the current insurance design that replies on localized administration and patients paying services up-front and requesting reimbursement later. To improve the equity in social insurance benefits between migrants and non-migrants and among migrants, policies that promote the insurance portability across regions and immediate reimbursement are warranted, while at the same time efforts should be done to control inflation of health care expenditures and to prevent inverse government subsidies from the regions that have more out-migration to regions that have in-migration.

Strengths and limitations of this study

- We aim to generate new evidence on the continuously changing health

For peer review only - http://bmjopen.bmj.com/site/about/guidelines.xhtml

insurance system in China, and provide policy implications for other developing countries thriving to achieve universal health coverage under rapid urbanization.

- Data are from the 2014 nationally representative cross-sectional sample of internal migrants in China, and a two-part model is used.
- The sensitivity analyses are conducted on total sample and two subsamples to get robust results.
- The outcomes related with reimbursement were self-reported, which may lead to measurement bias.
- Health status may influence health care utilization as well as whether a person chooses to migrate.

Keywords: migrant, health insurance, health equity, benefits, China

For peer review only - http://bmjopen.bmj.com/site/about/guidelines.xhtml

INTRODUCTION

Over the past decades, China has been experiencing the most rapid urbanization and internal migration[1]. Domestic (internal) migrants who lived outside their place of origin reached 245 million in 2016[2]. However, migrants faced huge barriers to obtain health insurance in the places where they lived[3]. Research has shown that migrants were less likely to be covered by social health insurance than permanent residents[4]. Although China established a nationwide social health insurance system, this system was quite fragmented and administrated locally, leading to large variation in insurance benefits across health insurance programs and regions. The unequal benefits in insurance might be amplified among the migrant population for the following reasons[5].

Firstly, the social health insurance system consisted of three separate insurance programs designed based on citizen's residence registration status and employment status[4]: the New Rural Cooperative Medical Scheme (NCMS) covering the registered rural residents, the Urban Employee Basic Medical Insurance (UEBMI) covering urban employees only, and the Urban Resident Basic Medical Insurance (URBMI) covering urban non-working residents[5-7]. UEBMI was jointly financed by employers and employees, while URBMI and NCMS were financed mostly by general taxes in addition to individual's premium. Subsidies from government accounted for up to 70% of the URBMI and NCMS funds[7]. Among the three insurance programs, UEBMI offers the most generous benefit package that provides reimbursement for both outpatient and inpatient services with high reimbursement rates, whereas URBMI and NCMS mainly reimburse for inpatient services[8]. Additionally, UEBMI and URBMI covered more than 2100 drugs, almost twice the coverage of NCMS. To promote urbanization, the Chinese government issued policies to allow migrants to enroll in UEBMI or URBMI depending upon their employment status[9], and thus migrants may be enrolled in any of the three insurance programs. This may result in the variation in insurance benefits among migrants.

Secondly, the social health insurance programs are administrated and financed by local county or city government [10]. Each county or city designs its own benefit package which mainly covers health services delivered within the county / city, and generally does not reimburse health services delivered outside of the county / city[8]. Even if some counties / cities cover health services provided outside of the county / city, lack of insurance portability across regions could create an additional barrier for insurance

For peer review only - http://bmjopen.bmj.com/site/about/guidelines.xhtml

enrollees to receive the benefits. Migrants may enroll in health insurance at one place, but move to another place and receive health services at this new place, which thus results in separation between the location of health care use and that of health insurance coverage. For many internal migrants, they need to go back to their place of origin to get insurance reimbursement.

Previous benefit analysis among general population showed that government subsidies for social health insurance were pro-rich for both inpatient and outpatient services from 2003 to 2013 in China, although inequity in benefit distribution had been narrowed[11-13]. Evidence from URBMI also showed that the lower-income groups benefited less than the higher-income groups between 2007 and 2011[14]. Huang's study further pointed out that with the fragmented feature and increased benefit disparities, social health insurance not only reinforced the existing rural-urban inequity, but also generated a new inequity between urban residents and migrants who lived in urban areas[7]. The only one study focusing on migrant workers presented that enrollment in UEBMI, URBMI or NCMS did not significantly increase health care utilization or reduce out-of-pocket health care expenditures[15]. In addition to income inequity, there is a unique contributor to health inequity among migrants – scope of migration – that where they migrated to. However, no literature has focused on the benefit distribution of social health insurance by scope of migration for the huge migrant population in China.

Using the 2014 China National Internal Migrants Dynamic Monitoring Survey, we conducted a benefit analysis of social health insurance among a representative sample of migrants in China. This study was the first to assess the benefit distribution by the geographic scope of migration and health insurance programs. We aimed to generate new evidence on the continuously changing health insurance system in China, and provide policy implications for other developing countries thriving to achieve universal health coverage under rapid urbanization.

METHODS

Data and Study Design

Data used for this analysis were from the 2014 China National Internal Migrant Dynamic Monitoring Survey. The survey was conducted by the National Health and Family Planning Commission of China in May 2014. This was a national cross-

For peer review only - http://bmjopen.bmj.com/site/about/guidelines.xhtml

sectional survey representing 15-59 year-old internal migrants who have lived in a city of new residence for more than one month but do not have a "Hukou" of the city (registered resident certificate).

In this survey, a stratified multi-stage random sampling method by Probability Proportional to Size (PPS) was employed, and the annual national data on internal migrants from each province in 2013 was considered as the basic sampling frame. A total of 348 cities from 32 provincial units in China were surveyed. Within each city, townships were randomly selected and followed by neighborhoods using the PPS. And then, in each neighborhood, 20 internal migrants were randomly selected to participate in the survey, finally reaching a total sample of 200,937 respondents. Face-to-face interview was conducted by trained interviewers, using a structured questionnaire. The informed consent was sought from the study respondents.

Questionnaires included demographic information and family structures, socioeconomic status, migration characteristics, health insurance, health care services, and family planning services. In this study, we focused on internal migrants who used inpatient care in the city of new residence during the previous year of the survey, and analyzed the benefits they received from their social health insurance. Thus, our sample comprised internal migrants who used inpatient care services in the city of new residence and had social health insurance, with a sample size of 1165 in total.

Patient and public involvement

No patient involved.

Measurements

In this analysis, benefits of health insurance were measured using three outcomes: the probability of receiving reimbursements from social health insurance, total amounts of reimbursement received, and the percentage of reimbursements of total health care expenditures (reimbursement ratio). In the survey, we identified the first outcome by a multiple-choice question "where did you receive reimbursement for your last hospitalization in this year", answers including: allowance from NCMS, allowance from UEBMI, the employer, the NCMS office, the local health centers, the commercial insurance, allowance from the Family planning operation, the Family planning operation, and else. We recognized those who answered only "the commercial insurance" or "else" or both "the commercial insurance" and "else" as receiving no

For peer review only - http://bmjopen.bmj.com/site/about/guidelines.xhtml

reimbursement from social health insurance. We identified the second outcome by the question "how much reimbursement did you receive from social health insurance. We identified the last outcome by the question for the second outcome and the question "how much did you cost in total".

Our primary predictors of interest were social health insurance programs and the geographic scope of migration. Social health insurance programs included UEBMI, URBMI and NCMS. The geographic scope of migration was categorized into three subgroups: migration across counties but within a city, migration across cities but within a province, and migration across provinces (Under China's administrative division, a county is smaller than a city).

Controlled variables included demographic characteristics, socioeconomic status, other migration characteristics, and the facility level for hospitalization. Demographic characteristics included gender, age and marital status. Marital status was measured by a binary variable indicating whether the respondent was married or not married (e.g. widowed, divorced or never married). Socioeconomic status was measured by educational attainment, monthly household income per capita, whether the respondent had a job, whether the respondent had rural "Hukou", and whether the respondent lived in urban areas. Educational attainment was categorized into four subgroups: primary school and below, junior high school, senior high school, and college degree and above. "Hukou" represents the record in the residency registration system in China; people can be registered as having either a rural or urban "Hukou" at birth and cannot be easily changed throughout lifetime[16]. Other migration characteristics were measured by reasons for and duration of migration. The reasons for migration included seeking jobs, family members following them to migrate or other reasons. Migration duration was categorized into four groups: less than one year; one to five years; five to ten years; and ten years and above. Finally, the facility level for hospitalization included primary care facility, secondary hospital, tertiary hospital, and private hospital.

Statistical analysis

We first described the general characteristics of our study sample. Chi-square test and one-way variance analysis were used to compare the differences of the probability

For peer review only - http://bmjopen.bmj.com/site/about/guidelines.xhtml

of receiving reimbursement, the amount and ratio of reimbursement received according to the geographic scope of migration.

Since there were many "zero observations" - patients who used inpatient care but received no reimbursement, we used the two-part model to estimate the benefits migrants received from the social health insurance, which can be expressed as follows:

$$\Pr\left[(\text{Reimburse amount})_i > 0 \;/\; (\text{Migrantion scope})_i\right] = \Phi[\beta * (\text{Migrantion scope})_i + \eta * X_i + \text{e}_i] \qquad (1)$$

$$log[(\text{Reimburse amount})_i / \; (\text{Reimburse amount})_i > 0, (\text{Migration scope})_i, \text{X}_i] = \theta * (\text{Migrantion scope})_i + \gamma * X_i + \psi_i \qquad (2)$$

$$(\text{Reimburse ratio})_i / \; (\text{Reimburse amount})_i > 0, (\text{Migration scope})_i, \text{X}_i = \varepsilon * (\text{Migrantion scope})_i + \delta * X_i + \alpha_i \qquad (3)$$

Where $(Reimburse\ amount)_i$ and $(Reimburse\ ratio)_i$ are the reimbursement amount and reimbursement ratio received by individual i. $(Migrantion\ scope)_i$ is a set of dummies representing migration scopes of individual i, and migrants who migrated across counties within a city is taken as the reference group. The parameter β, θ and ε, the key coefficients of interest, identify the association between migration scope and the probability of receiving reimbursement, and also the amount and ratio of reimbursement conditional on reimbursement received, respectively. Xi is a vector of control variables including social health insurance coverage, demographic characteristics, socioeconomic status, other migration characteristics, and the facility level for hospitalization as mentioned above.

The above two-part model assumes that the benefit migrants received from the social health insurance is determined by two separate decision making process: equation (1), the 'participation equation', captures the fundamental difference between the respondents who received reimbursements from social health insurance and those who did not; as the 'intensity equation', equation (2) or equation (3) characterizes the determinants of the amount and the ratio of reimbursement received among those who actually received reimbursements. In equation (2) and equation (3), the amount and the ratio of the reimbursement fits the Gamma distribution, and the logarithm transformation was taken on the amount of the reimbursement to reduce the impact of extreme values. Following the previous studies [e.g. Jan pan, Sen Tian, Qin Zhou and Wei Han (2016)[614], we estimated equation (1) with the Probit Model, and equation (2)

For peer review only - http://bmjopen.bmj.com/site/about/guidelines.xhtml

and equation (3) with the Generalized Linear Model (GLM), respectively. Marginal effects with standard errors were reported.

All of the analyses were conducted for the total sample, rural social insurance sample (the NCMS subsample), and urban social insurance sample (the URBMI & UEBMI subsample), respectively. All analyses were performed using STATA 12.0 (StataCorp LP, College Station, TX, USA).

Results

Characteristics of the study sample

Table 1 presents the descriptive statistics for our study sample. Of the 1165 respondents, 66.70% enrolled in NCMS, and 23.00% and 10.30% enrolled in UEBMI and URBMI respectively. The average expenditures per inpatient stay were 10,366 Chinese Yuan (=1,567 US dollar), and there was a small difference (about 500 Yuan , P=0.643) of inpatient expenditure between the NCMS subsample and URBMI & UEBMI subsample. 66.78% of respondents who used inpatient care received reimbursement from social health insurance, with 60.49% for NCMS enrollees and 79.38% for URBMI & UEBMI enrollees. Among the respondents who received reimbursement, the average amount and ratio of the reimbursement received were 5,506 Yuan (=832 US dollar) and 46.77%. The average amount and ratio of the reimbursement received for NCMS enrollees were much smaller than those for URBMI & UEBMI enrollees.

The average age and monthly household income per capita of the respondents was 38 years old and 2,256 Yuan. Less than half of the respondents were female. Most of them were married (89.27%), had education level of high school or below (89.70%), had rural "Hukou" (87.81%) and owned a job (79.57%), and lived in urban areas (69.36%). Nearly half of the respondents migrated across provinces, while those who migrated across cities but within a province and those who migrated across counties but within a city were 28.76% and 25.41% respectively. A total of 84.21% respondents migrated for better job opportunities, and 87.38% respondents had lived in the city of new residence for more than one year. Most of respondents (80.51%) chose inpatient care at secondary and tertiary hospitals instead of primary care facilities.

For peer review only - http://bmjopen.bmj.com/site/about/guidelines.xhtml

Table 1 Characteristic of the study sample, n (%)

Variables	Total sample (N=1165)	NCMS subsample (N=777)	URBMI&UEBMI subsample (N=388)
Total expenditure per inpatient stay (Yuan) *	10366.05 (±17549.73)	10197.45 (±18374.85)	10704.99 (±15778.28)
Probability of receiving reimbursement	778(66.78)	470(60.49)	308(79.38)
Reimbursement amount received (Yuan) *	5506.16 (±9761.79)	5128.44 (±10838.10)	6049.14 (±7952.00)
Reimbursement ratio received (%) *	46.77(±19.91)	39.41(±17.86)	57.35(±17.87)
Social health insurance programs [a]			
NCMS	777(66.70)	-	-
URBMI	120(10.30)	-	120(30.93)
UEBMI	268(23.00)	-	268(69.07)
Age (Years) *	37.65(±9.75)	37.79(±9.80)	37.37(±9.65)
Female	532(45.67)	361(46.46)	171(44.07)
Married	1040(89.27)	705(90.73)	335(86.34)
Education attainment			
Primary school and below	279(23.95)	218(28.06)	61(15.72)
Junior high school	554(47.55)	416(53.54)	138(35.57)
Senior high school	212(18.20)	118(15.19)	94(24.23)
College and above	120(10.30)	25(3.22)	95(24.48)
Monthly household income per capita (Yuan) *	2256.43(±2092.85)	1999.55(±1687.47)	2770.86(±2658.26)
Having any job	927(79.57)	610(78.51)	317(81.70)
Rural Hukou	1023(87.81)	777(100.00)	267(68.81)
Living in urban area	808(69.36)	502(64.61)	306(78.87)
Geographic scope of migration			
Across counties within a city	296(25.41)	216(27.80)	80(20.62)
Across cities within a province	335(28.76)	214(27.54)	121(31.19)
Across provinces	534(45.84)	347(44.66)	187(48.20)
Reasons of migration			
Seeking jobs	981(84.21)	642(82.63)	339(87.37)
Family members following migrants	146(12.53)	115(14.80)	31(7.99)
Other reasons	38(3.26)	20(2.57)	18(4.64)
Migration duration (Years)			
0~1	147(12.62)	117(15.06)	30(7.73)
1~5	484(41.55)	305(39.25)	179(46.13)
5~10	272(23.35)	181(23.29)	91(23.45)
10 and above	262(22.49)	174(22.39)	88(22.68)
Facility level of hospitalization			
Primary care facility	131(11.24)	90(11.58)	41(10.57)

For peer review only

For peer review only - http://bmjopen.bmj.com/site/about/guidelines.xhtml

Secondary hospital	400(34.33)	282(36.29)	118(30.41)
Tertiary hospital	538(46.18)	340(43.76)	198(51.03)
Private hospital	96(8.24)	65(8.37)	31(7.99)

Note: * mean (± Standard Deviation).

a. NCMS: New Rural Cooperative Medical Scheme; URBMI: Urban Resident Basic Medical Insurance; UEBMI: Urban Employee Basic Medical Insurance.

Table 2 summarizes the total expenditures per inpatient stay, the probability of benefiting from the social health insurance. It also presents that among the benefit recipients, the amount and ratio of reimbursement received according to the geographic scope of migration. The univariate analysis showed that the broader the migration scope, the lower the probability that migrants would receive reimbursements; but among those who received reimbursements, those who migrated across cities or across provinces received the larger amounts of reimbursement than those who migrated within a city. There was no significant difference in total expenditure and reimbursement ratio by the geographic scope of migration.

Table 2 Benefit distribution of social health insurance by geographic scope of migration

Variables	Across counties within a city	Across cities within a province	Across provinces	P
Total expenditure per inpatient stay (Yuan)	10326.81 (14079.35)	14458.96 (27139.10)	12771.68 (20428.78)	0.354
Probability of receiving reimbursement (%)	81.76 (38.69)	69.85 (45.96)	56.55 (49.62)	<0.001
Reimbursement amount received (Yuan)	4402.12 (6541.01)	6706.32 (13681.56)	5495.89 (8105.80)	0.056
Reimbursement ratio received (%)	44.75 (18.17)	47.76 (20.51)	47.72 (20.79)	0.195

Note: Standard Deviations are reported in parentheses.

Association between insurance programs, migration scope and benefit of social health insurance

Table 3 reports the association between insurance programs, migration scope, other factors and benefit of social health insurance, estimated from a two-part model.

For peer review only

For peer review only - http://bmjopen.bmj.com/site/about/guidelines.xhtml

Compared with NCMS enrollees, URBMI or UEBMI enrollees were more likely to receive reimbursement, and among the benefit recipients, urban insurance enrollees received larger reimbursement amount and ratio. The probability of receiving reimbursement for UEBMI enrollees was 37.5% (P<0.01) higher than that for the NCMS enrollees. Among insurance benefit recipients, UEBMI enrollees received 42.8% (P<0.01) more reimbursement amount and 20.1% (P<0.01) higher reimbursement ratio than NCMS enrollees.

According to the association between insurance benefit and migration scope, the geographic scope of migration significantly reduced the probability of receiving reimbursement and the reimbursement ratio, but increased the reimbursement amounts they received. Specifically, the probability of receiving reimbursement for those who migrated across cities and provinces was 14.7% and 26.0%, respectively, lower than those who migrated within a city (P<0.01). However, they received 33.4% and 27.2% higher amount of reimbursement than those who migrated within a city (P<0.01). And those who migrated across provinces had the lowest reimbursement ratio (P<0.10).

In addition, there was no significant difference in insurance benefits by age, gender, marriage status, education, Hukou status, and migration duration. Income had no significant influence on the probability of receiving reimbursement and reimbursement ratio, but significantly increased the reimbursement amount. Having jobs significantly decreased the probability and amount of receiving reimbursement, whereas living in urban areas significantly increased the probability of receiving reimbursement by 6.3% than living in suburban areas. Compared with migration for seeking jobs, family members following migrants significantly increased the reimbursement amount and ratio. The higher the level of health care facility, the greater probability and amount of receiving reimbursement, but the lower the reimbursement ratio.

Table 3 Association between insurance programs, migration scope, other factors and benefit of social health insurance

Variables	Probit Model		Generalized Linear Model			
	Probability of getting reimbursement		Reimbursement amount		Reimbursement ratio	
	Coefficient	S.E.	Coefficient	S.E.	Coefficient	S.E.
Social health insurance (referred to NCMS)						

For peer review only - http://bmjopen.bmj.com/site/about/guidelines.xhtml

URBMI	0.021	0.049	0.222	0.136	0.147***	0.031
UEBMI	0.375***	0.044	0.428***	0.094	0.201***	0.023
Age (years)	0.010	0.012	0.026	0.030	0.006	0.006
Age^2 (years)	0.000	0.000	0.000	0.000	0.000	0.000
Female	0.024	0.031	-0.004	0.078	0.018	0.016
Married	0.063	0.051	-0.037	0.146	0.010	0.032
Education attainment (referred to primary school and below)						
Junior high school	0.039	0.038	0.108	0.099	0.014	0.020
Senior high school	0.081	0.050	0.014	0.125	0.027	0.026
College and above	-0.038	0.068	-0.015	0.173	0.032	0.039
Monthly household income per capita (referred to 1st quintile)						
2nd quintile	0.062	0.048	0.328***	0.124	-0.029	0.026
3rd quintile	-0.033	0.051	0.014	0.139	-0.041	0.028
4th quintile	0.047	0.049	0.179	0.124	-0.031	0.026
5th quintile	0.029	0.053	0.336**	0.137	-0.023	0.030
Having any job	-0.070*	0.042	-0.247**	0.109	0.010	0.022
Rural hukou	0.088	0.053	-0.106	0.126	0.000	0.031
Living in urban area	0.063**	0.032	-0.012	0.088	0.004	0.017
Geographic scope of migration (referred to migration across counties within a city)						
Across cities within a province	-0.147***	0.041	0.334***	0.093	-0.019	0.019
Across provinces	-0.260***	0.038	0.272***	0.093	-0.032*	0.019
Reasons of migration (referred to Seeking jobs)						
Family members following migrants	-0.007	0.050	0.553***	0.137	0.047*	0.027
other reasons	-0.025	0.087	-0.224	0.207	0.024	0.048
Migration duration (referred to 0~)						
1~	-0.014	0.045	0.078	0.125	-0.032	0.026
5~	0.017	0.051	0.162	0.135	-0.022	0.028
10~	-0.011	0.052	0.085	0.140	-0.040	0.029
Facility level of hospitalization (referred to primary care facility)						
Secondary hospital	-0.031	0.048	0.603***	0.139	-0.046	0.032
Tertiary hospital	0.083*	0.048	1.065***	0.135	-0.083***	0.031
Private hospital	-0.156**	0.063	0.092	0.188	-0.027	0.043
Observations	1165		663		663	

Note: ***, ** and * denote statistical significance at 1, 5 and 10% level, respectively. S.E.: Standard Error. NCMS: New Rural Cooperative Medical Scheme; URBMI: Urban Resident Basic Medical Insurance; UEBMI: Urban Employee Basic Medical Insurance.

Considering the differences in reimbursement policy between NCMS and urban health insurance, we further conducted the above regressions among the subsamples of

For peer review only

For peer review only - http://bmjopen.bmj.com/site/about/guidelines.xhtml

NCMS enrollees and URBMI & UEMBI enrollees (Table 4). The relationships between migration scope and the probability of receiving reimbursement and reimbursement amount did not change, while it differed in its relationship with reimbursement ratio. Those who migrated more broadly had a significantly lower reimbursement ratio among NCMS enrollees, but had a significantly higher ratio among URBMI & UEMBI enrollees.

Table 4 Association between migration scope and benefit by social health insurance programs

Variables	Probit Model		Generalized Linear Model			
	Probability of getting reimbursement		Reimbursement amount		Reimbursement ratio	
	Coefficient	S.E.	Coefficient	S.E.	Coefficient	S.E.
**NCMS subsample**						
Geographic scope of migration (referred to migration across counties within a city)						
Across cities within a province	-0.139***	0.052	0.410***	0.120	-0.044*	0.023
Across provinces	-0.333***	0.048	0.282**	0.118	-0.055**	0.024
**URBMI & UEBMI subsample**						
Geographic scope of migration (referred to migration across counties within a city)						
Across cities within a province	-0.135**	0.061	0.339**	0.154	0.073**	0.033
Across provinces	-0.118*	0.061	0.285*	0.152	0.039	0.033

Note: All models included confounding factors in Table 3. ***, ** and * denote statistical significance at 1, 5 and 10% level, respectively.

S.E.: Standard Error. NCMS: New Rural Cooperative Medical Scheme; URBMI: Urban Resident Basic Medical Insurance; UEBMI: Urban Employee Basic Medical Insurance.

Reasons for not receiving reimbursement from social health insurance

We further investigated the reasons why migrants did not receive reimbursement from their social health insurance. Figure 1 showed that the need or plan to go back to hometown to get reimbursement was the main reason for not getting reimbursement, accounting for 66.5%, followed by a lack of knowledge about the reimbursement process (16.7%) and the policy coverage issues (10.6%). Figure 2 further compares the proportion of not receiving reimbursement due to the need or plan to go back to

For peer review only - http://bmjopen.bmj.com/site/about/guidelines.xhtml

hometown by migration scope. The broader the migration scope, the higher the likelihood that migrants did not receive reimbursement because that they must get reimbursement later from their hometowns.

Figure 1. Reasons not getting reimbursement from social health insurance (on the left)

Figure 2. Proportion of not getting reimbursement due to the need or plan to go back to hometown for reimbursement, by geographic scope of migration (on the right)

Discussion

Using recent data from the China National Internal Migrants Dynamic Monitoring Survey, this study documented the benefit distribution of social health insurance among internal migrates. Among migrants who utilized inpatient care in the past year, only 67% received reimbursements from their social health insurance, and the reimbursement amount only accounted for 47% of the total expenditure per inpatient stay. There were large disparities by health insurance programs and migration scope. The broader the migration scope, the lower the probability of receiving reimbursement and the reimbursement ratio, but the higher the reimbursement amount they received.

Comparing this finding with other studies we found there was an inequity in insurance benefits between migrants and non-migrant population. The probability of receiving reimbursement and the reimbursement ratio for migrants were far smaller than those for the general population. Only 60% of migrants who were NCMS enrollees received reimbursement from NCMS, which was 30% lower than that for general NCMS enrollees (91.1% in 2013), whereas the reimbursement ratio among this group of migrants was 10% lower than that among general NCMS enrollees (39.4% vs. 50.1% in 2013)[17]. For URBMI & UEBMI enrollees, the probability of receiving reimbursement among migrants was about 10% lower than that among general population (79.4% vs. 95.3% for UEBMI and 88.7% for URBMI enrollees), although small difference existed in the reimbursement ratio between the two groups (around 60%) [17]. A previous study also pointed out that migrants only partially benefited from health insurance coverage[15].

The results should be understood in the China specific context. There were several challenges for migrants to get insurance benefits under the current insurance policy

For peer review only - http://bmjopen.bmj.com/site/about/guidelines.xhtml

design. In China, migrants faced more challenges of getting insurance reimbursement than non-migrants. There have been two common approaches to reimburse health care services (immediate reimbursement and later reimbursement)[18]. Immediate reimbursement means that the insured patient gets reimbursement immediately for the treatment and only pay out-of-pocket for the copay or coinsurance rate, whereas later reimbursement means that the insured patient pays the total expenditures out-of-pocket up-front and gets reimbursement later from their health insurance[18]. Local residents usually get reimbursement immediately, but migrants in general receive reimbursement later as they must travel back to their hometown where they enroll in social health insurance to receive reimbursement. Our study showed that up to 22% of migrants reported that did not get reimbursement because they needed to get reimbursement from their hometowns. In addition, research has shown that many services were not reimbursed and the reimbursement process was much more complex for migrants than for local residents[19]. One study showed that more migrants were treated in a hospital that outside of the NCMS designated network than local residents, and thus their healthcare use were less likely to be covered by NCMS [19].

Our findings also showed inequity in insurance benefits among migrants by migration scope. Although the scope of migration was associated with larger reimbursement amount per inpatient stay, it was significant associated with a lower probability of receiving reimbursement and a lower reimbursement ratio. In China, all three social health insurance programs were administered, financed and operated by local county or city governments. Each county or city designed its own benefit packages and made the benefit localized[8 20], which limited individual coverage choices outside of the local region. This poses a challenge for internal migrants who typically use health care in the city of new residence, but may enroll in health insurance at their hometown according to their residence ("Hukou") status. The separation between where health care is received and where health insurance is administrated provided an additional hurdle for the internal migrants to receive benefits from their social health insurance. It became even more difficult when they lived far away from their hometowns. The localized administration of social health insurance and the later reimbursement approach contributed jointly to the inequity in benefit coverage for migrants[21]. To resolve this inequity, policies that promote the portability of insurance across regions

For peer review only - http://bmjopen.bmj.com/site/about/guidelines.xhtml

and link health information to ease the reimbursement approach should be encouraged[21-22].

Another important finding of this study was that migrants who got reimbursement received larger reimbursement amounts if they migrated more broadly. Compared to migration within a city, migration across cities or across provinces was significantly associated with 30% higher reimbursement amount per inpatient stay. This mainly attributed to higher health care costs in larger cities and more affluent regions where migrants who migrated more broadly like to locate. However, these reimbursements were mainly paid by health insurance funds in smaller counties / cities which were less affluent. This may pose financial difficulties for local governments in those out-migration regions, as if the current barriers to reimbursement were to be removed, there would have been larger amounts of insurance funds flowing into the health care system in more prosperous in-migration regions, which would worsen the already skewed regional inequity in economic development and health. Therefore, the inverse subsidies of health insurance funds from the less-developed out-migration regions to the highly-developed in-migration regions became an ongoing challenge and policy dilemma for countries like China that experience a mass domestic migration. Strategies that control health expenditures inflation and allow central government to redistribute welfare funding to less-developed regions may be warranted to address the possible financial difficulties faced by out-migration regions[5].

In addition to the above inequity related with migration. There were two other types of inequity in benefits due to the fragmented social insurance system and income inequity. Although China has almost achieved universal insurance coverage through social health insurance expansion[5], migrants who enrolled in UEBMI and URBMI benefited more than those who enrolled in NCMS [8 23]. One national study found that among general population, UEBMI enrollees had a higher benefit level than those covered by URBMI or NCMS[24]. Even for the same condition - tuberculosis inpatient care- the reimbursement rate was the highest for UEBMI enrollees, followed by URBMI, and NCMS enrollees in 2012[23.] Income inequity also explains some of the benefit inequities. Among the benefit recipients, we found that migrants with higher income received greater reimbursement amount, which was consistent with prior studies among the general population[11 14 25]. On average, the higher-income group

For peer review only - http://bmjopen.bmj.com/site/about/guidelines.xhtml

tended to have higher inpatient expenditure, and it was not surprising that they also received greater reimbursement amount[26].

This study contained some limitations. First, the outcomes related with reimbursement were self-reported, which may lead to measurement bias. Future research will use health insurance claims data to minimize this bias. Second, health status may influence health care utilization as well as whether a person chooses to migrate. Studies have detected the healthy migrant effect for internal migrants in China, showing that healthier people were more likely to migrate and to move farther away from home[27]. Unfortunately, there was no measurement on health status of migrants in this dataset. Third, we could not accurately distinguish the administration location of social health insurance, which may affect the insurance benefit for internal migrants. To reduce this bias, we conducted the analysis separately on two subsamples of NCMS enrollees and UEBMI & URBMI enrollees.

Conclusions

This study has important policy implications for China and other developing countries that experience rapid urbanization and internal migration. The broader the migration scope, the lower the probability of receiving reimbursements from the social insurance and the reimbursement ratio; but among those who received reimbursements, the broader the migration scope, the larger amounts they were reimbursed for health care use. We attributed this unequal benefit distribution to the cur-rent insurance design that replies on localized administration and patients paying services up-front and requesting reimbursement later. To improve the equity in social insurance benefits between migrants and non-migrants and among migrants, policies that promote the insurance portability across regions and immediate reimbursement are warranted, while at the same time efforts should be done to control inflation of health care expenditures and to prevent inverse government subsidies from the regions that have more out-migration to regions that have in-migration.

Contribution Statement

ZH designed the study and drafted the manuscript. HW conducted the literature

For peer review only - http://bmjopen.bmj.com/site/about/guidelines.xhtml

review, analyzed the data, and contributed to the manuscript writing. DL and DZ revised the manuscript. All authors read and approved the final manuscript being submitted.

Acknowledgments

We thank the National Health and Family Planning Commission of China for providing the data.

Competing Interests

None.

Funding

This work was supported by the National Natural Science Foundation of China (No. 71874034) and the National Key R&D Program of China (No. 2018YFC1312600 and 2018YFC1312604).

Ethics approval

The data was a public data, organized by the National Health and Family Planning Commission of China.

Data availability statement

The data used in this paper were provided by the National Health and Family Planning Commission of China. We signed a legally binding agreement with the Commission that we would not share the original data to any third parties. Although researchers who are interested can apply to get access to the data at: http://www.nhc.gov.cn/rkjcyjtfzs/zcwj2/201510/db6c46c4ef114f1092bb2c4d85846e0f.shtml and Email: ldrkzxsj@163.com, it is up to the National Health and Family Planning Commission to make the final decision.

For peer review only

For peer review only - http://bmjopen.bmj.com/site/about/guidelines.xhtml

References

1 P Gong, S Liang, EJ Carlton, et al. Urbanisation and health in China. *The Lancet* 2012;379(9818):843-52.

2 National Bureau of Statistics of the People's Republic of China. Statistical Communique on National Economic and Social Development in 2016. In, 2017.

3 JX Fan, M Wen, L Jin, G Wang. Disparities in Healthcare Utilization in China: Do Gender and Migration Status Matter? *J Fam Econ Iss* 2013;34(1):52-63.

4 Y Jin, Z Hou, D Zhang. Determinants of Health Insurance Coverage among People Aged 45 and over in China: Who Buys Public, Private and Multiple Insurance. *Plos One* 2016;11(8):e161774.

5 Q Meng, H Fang, X Liu, B Yuan, J Xu. Consolidating the social health insurance schemes in China: towards an equitable and effi cient health system. *The lancet* 2015;386(10002):1484-92.

6 Z Hou, E Van de Poel, E Van Doorslaer, B Yu, Q Meng. Effects of NCMS Coverage on Access to Care and Financial Protection in China. *SSRN Electronic Journal* 2012.

7 X Huang. Expansion of Chinese Social Health Insurance: Who Gets What, When and How? *J Contemp China* 2014;23(89):923-51.

8 Q Wang, D Zhang, Z Hou. Insurance coverage and socioeconomic differences in patient choice between private and public health care providers in China. *Soc Sci Med* 2016;170:124-32.

9 Ministry of Human Resources and Social Security of the People's Republic of China. Interim Measures for the Transferability of Basic Medical Insurance Account among Internal Migrants. In, 2009.

10 M Ramesh, X Wu, AJ He. Health governance and healthcare reforms in China. *Health Policy Plann* 2014;29(6):663-72.

11 M Chen, G Fang, L Wang, et al. Who Benefits from Government Healthcare Subsidies? An Assessment of the Equity of Healthcare Benefits Distribution in China. *Plos One* 2015;10(3):e119840.

12 M Chen, AJ Palmer, L Si. Assessing equity in benefit distribution of government health subsidy in 2012 across East China: benefit incidence analysis. *Int J Equity Health* 2016;15:15.

13 L Si, M Chen, AJ Palmer. Has equity in government subsidy on healthcare improved in China? Evidence from the China's National Health Services Survey. *Int J Equity Health* 2017;16(1):6.

14 J Pan, S Tian, Q Zhou, W Han. Benefit distribution of social health insurance: evidence from china’

For peer review only - http://bmjopen.bmj.com/site/about/guidelines.xhtml

s urban resident basic medical insurance. *Health Policy Plann* 2016;31(7):853-59.

15 X Qin, J Pan, GG Liu. Does participating in health insurance benefit the migrant workers in China? An empirical investigation. *China Econ Rev* 2014;30:263-78.

16 Z Zhang, DJ Treiman. Social origins, hukou conversion, and the wellbeing of urban residents in contemporary China. *Soc Sci Res* 2013;42(1).

17 National health commission of the People's Republic of China. An Analysis Report of National Health Services Survey in China, 2013. Beijing: Peking Union Medical College Press 2015.

18 H Zhong. Effect of patient reimbursement method on health-care utilization: evidence from China. *Health Econ* 2011;20(11):1312-29.

19 P Qiu, Y Yang, J Zhang, X Ma. Rural-to-urban migration and its implication for new cooperative medical scheme coverage and utilization in China. *Bmc Public Health* 2011;11(1):520.

20 Y Zhao, B Kang, Y Liu, et al. Health insurance coverage and its impact on medical cost: observations from the floating population in China. *Plos One* 2014;9(11):e111555.

21 Q Gao, S Yang, S Li. Labor contracts and social insurance participation among migrant workers in China. *China Econ Rev* 2012;23(4).

22 KKF Lam, JM Johnston. Health insurance and healthcare utilisation for Shenzhen residents: a tale of registrants and migrants? *Bmc Public Health* 2012;12(1):868.

23 Y Pan, S Chen, M Chen, et al. Disparity in Reimbursement for Tuberculosis Care Among Different Health Insurance Schemes: Evidence From Three Counties in Central China. *Infect Dis Poverty* 2016;5(1):7.

24 L X, J WY. Empirical Study on the Benefit Equity of Essential Health Insurance System in China. *medicine and society* 2010;23(11):45-47. (Chinese)

25 S Yuan, C Rehnberg, X Sun, X Liu, Q Meng. Income related inequalities in New Cooperative Medical Scheme: a five-year empirical study of Junan County in China. *Int J Equity Health* 2014;13(1):1-15.

26 Z Zhou, J Gao, A Fox, et al. Measuring the equity of inpatient utilization in Chinese rural areas. *Bmc Health Serv Res* 2011;11(1):201.

27 Lijian, Qin, Yao, Lu. Healthy Migrant and Salmon Bias Hypotheses: A Study of Health and Internal Migration in China. *Soc Sci Med* 2014.

For peer review only

For peer review only - http://bmjopen.bmj.com/site/about/guidelines.xhtml

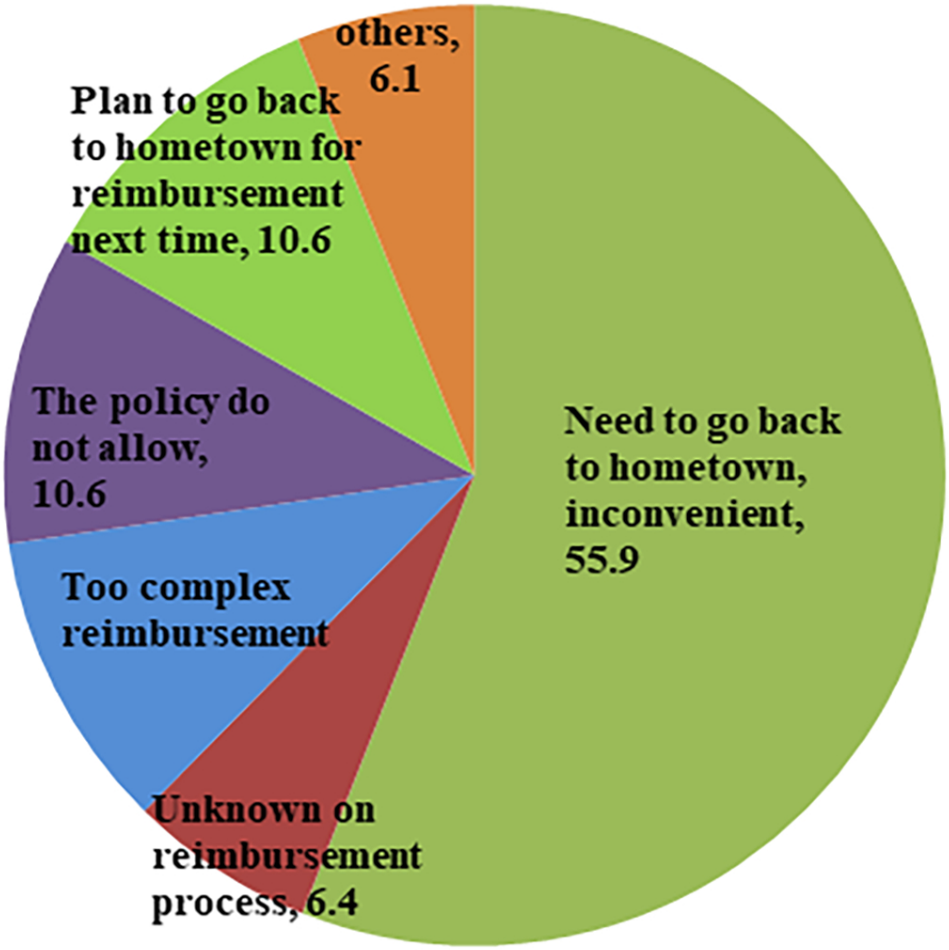

Figure 1. Reasons not getting reimbursement from social health insurance

228x242mm (118 x 118 DPI)

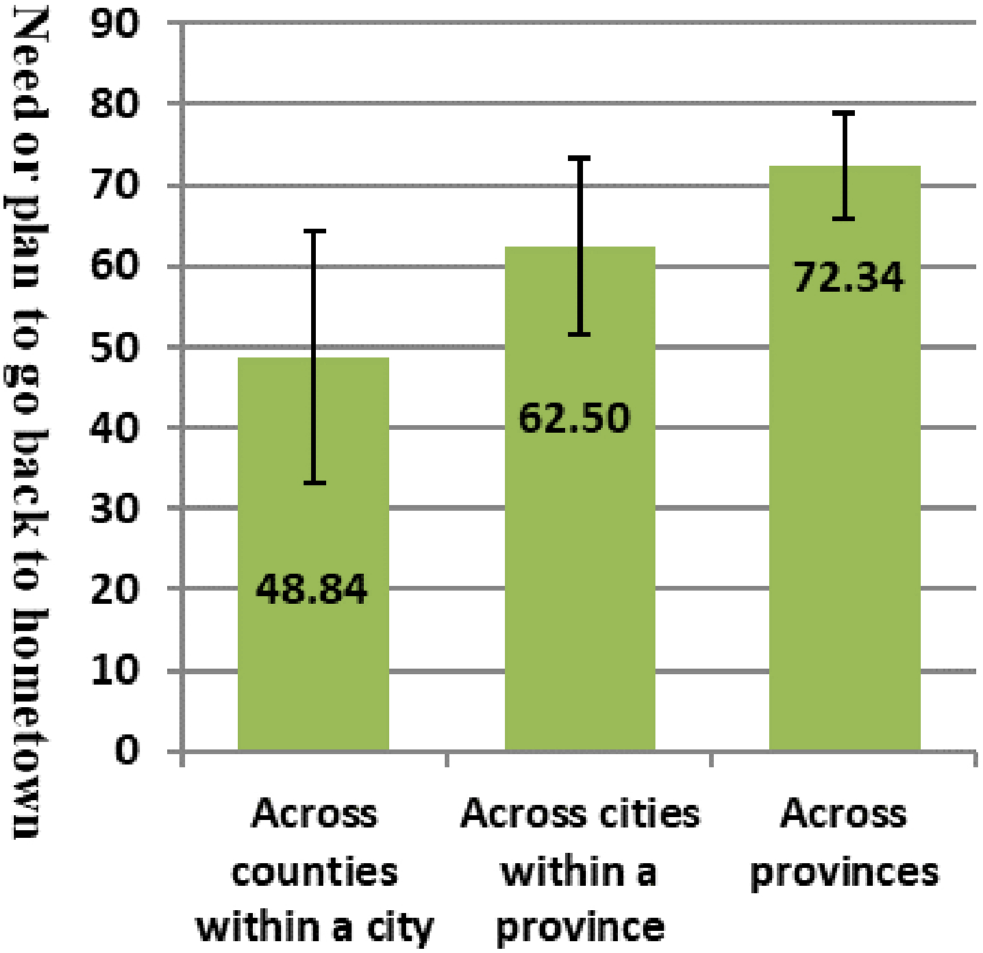

Figure 2. Proportion of not getting reimbursement due to the need or plan to go back to hometown for reimbursement, by geographic scope of migration

228x235mm (118 x 118 DPI)

BMJ Open

How does domestic migration pose a challenge in achieving equitable social health insurance benefits in China? a National Cross-Sectional Study

Journal:	*BMJ Open*
Manuscript ID	bmjopen-2021-060551.R1
Article Type:	Original research
Date Submitted by the Author:	13-May-2022
Complete List of Authors:	Wang, Haiqin; The International Peace Maternity & Child Health Hospital of China Welfare Institute, ; Fudan University School of Public Health, Liang, Di; University of California San Diego, Department of Family Medicine and Public Health Zhang, Donglan; University of Georgia, Department of Health Policy and Management, College of Public Health Hou, Zhiyuan; Fudan University
**Primary Subject Heading**:	Health policy
Secondary Subject Heading:	Health policy, Health economics
Keywords:	Health policy < HEALTH SERVICES ADMINISTRATION & MANAGEMENT, HEALTH SERVICES ADMINISTRATION & MANAGEMENT, Health economics < HEALTH SERVICES ADMINISTRATION & MANAGEMENT

SCHOLARONE™
Manuscripts

I, the Submitting Author has the right to grant and does grant on behalf of all authors of the Work (as defined in the below author licence), an exclusive licence and/or a non-exclusive licence for contributions from authors who are: i) UK Crown employees; ii) where BMJ has agreed a CC-BY licence shall apply, and/or iii) in accordance with the terms applicable for US Federal Government officers or employees acting as part of their official duties; on a worldwide, perpetual, irrevocable, royalty-free basis to BMJ Publishing Group Ltd ("BMJ") its licensees and where the relevant Journal is co-owned by BMJ to the co-owners of the Journal, to publish the Work in this journal and any other BMJ products and to exploit all rights, as set out in our licence.

The Submitting Author accepts and understands that any supply made under these terms is made by BMJ to the Submitting Author unless you are acting as an employee on behalf of your employer or a postgraduate student of an affiliated institution which is paying any applicable article publishing charge ("APC") for Open Access articles. Where the Submitting Author wishes to make the Work available on an Open Access basis (and intends to pay the relevant APC), the terms of reuse of such Open Access shall be governed by a Creative Commons licence – details of these licences and which Creative Commons licence will apply to this Work are set out in our licence referred to above.

Other than as permitted in any relevant BMJ Author's Self Archiving Policies, I confirm this Work has not been accepted for publication elsewhere, is not being considered for publication elsewhere and does not duplicate material already published. I confirm all authors consent to publication of this Work and authorise the granting of this licence.

For peer review only

How does domestic migration pose a challenge in achieving equitable social health insurance benefits in China? a National Cross-Sectional Study

Haiqin Wang[a, b#], Di Liang[b], Donglan Zhang[c], Zhiyuan Hou[b#*]

a. Administrative Office, The International Peace Maternity & Child Health Hospital of China Welfare Institute, 910 Heng Shan Road, Shanghai 200030, China
b. School of Public Health, NHC Key Laboratory of Health Technology Assessment, Fudan University, 138 Yi Xue Yuan Road, Shanghai 200032, China
c. Division of Health Services Research, Department of Foundations of Medicine, New York University Long Island School of Medicine, 101 Mineola Blvd, Mineola, NY, 11501, USA

[#] Co-first author, equally contribute to the article.

*Corresponding to Zhiyuan Hou, Ph.D., Associate professor,
School of Public Health, NHC Key Laboratory of Health Technology Assessment, Fudan University
Address: 138 Yi Xue Yuan Road, Shanghai 200032, China
Email: zyhou@fudan.edu.cn

Word count: 4375

For peer review only

For peer review only - http://bmjopen.bmj.com/site/about/guidelines.xhtml

Abstract

Objectives: To evaluate the benefit distribution of social health insurance among internal migrants in China.

Design: A national cross-sectional survey.

Setting: 348 cities from 32 provincial units in China.

Participants: 1165 internal migrants who used inpatient care services in the city of a new residence and had social health insurance.

Primary and secondary outcome measures: The probability of receiving reimbursements from social health insurance, the amounts and ratio of reimbursement received.

Results: Among migrants who utilized inpatient care in 2013, only 67% received reimbursements from social health insurance, and the reimbursement amount only accounted for 47% of the inpatient care expenditure. The broader the geographic scope of migration, the lower the probability of receiving reimbursement and the reimbursement ratio, but the higher the reimbursement amount. Specifically, the probability of receiving reimbursements for those who migrated across cities and provinces was 14.7% and 26.0%, respectively, lower than those who migrated within a city (P<0.01). However, they received 33.4% and 27.2% higher reimbursement amounts than those who migrated within a city (P<0.01). And those who migrated across provinces had the lowest reimbursement ratio (P<0.10).

Conclusions: The unequal benefit distribution may be attributed to the fragmented health insurance design that relies on localized administration and patients paying services up-front and requesting reimbursement later. To improve the equity in social insurance benefits, China has been promoting the portability of social health insurance and immediate reimbursement for inpatient care utilized across regions and a more integrated health insurance system. Efforts should also be made to control inflation of health care expenditures and prevent inverse government subsidies from out-migration regions to in-migration regions. This study has policy implications for China and other developing countries that experience rapid urbanization and internal migration.

For peer review only - http://bmjopen.bmj.com/site/about/guidelines.xhtml

Strengths and limitations of this study

- Data are from a 2014 nationally representative cross-sectional sample of internal migrants in China, and a two-part model is used.
- Sensitivity analyses are conducted on a total sample and two subsamples to get robust results.
- The outcomes related to insurance reimbursement were self-reported, leading to measurement bias.
- Health status may influence health care utilization as well as migration status.

Keywords: migrant, health insurance, health equity, benefits, China

For peer review only - http://bmjopen.bmj.com/site/about/guidelines.xhtml

INTRODUCTION

Over the past decades, China has been experiencing rapid urbanization and internal migration[1]. Domestic (internal) migrants who lived outside their place of origin reached 375million in 2020[2]. However, migrants faced huge barriers to obtaining health insurance in the places they lived[3-4]. Research has shown that migrants were less likely to be covered by social health insurance than permanent residents[4-5]. Although China established a national social health insurance system and made efforts to make the social health insurance system portable, this system was quite fragmented and administrated locally, leading to considerable variation in insurance benefits across health insurance programs and regions[6]. The unequal benefits in insurance might be amplified among the migrant population for the following reasons.

Firstly, the social health insurance system consisted of three separate insurance programs designed based on citizen's residence registration status and employment status[5]: the New Rural Cooperative Medical Scheme (NCMS) covering the registered rural residents, the Urban Employee Basic Medical Insurance (UEBMI) covering urban employees only, and the Urban Resident Basic Medical Insurance (URBMI) covering urban non-working residents[6-8]. UEBMI was jointly financed by employers and employees, while URBMI and NCMS were financed chiefly by general taxes in addition to individual premiums. Since 2016, integrating URBMI and NCMS to Urban-Rural Resident Basic Medical Insurance (URRBMI) was piloted in some provinces and gradually expanded nationally, although it is still administrated locally[9]. Subsidies from the government accounted for up to 70% of the URBMI and NCMS funds. Among the three insurance programs, UEBMI offers the most generous benefits package that provides reimbursement for both outpatient and inpatient services with high reimbursement rates, whereas URBMI and NCMS mainly reimburse for inpatient services[10]. To promote urbanization, the Chinese government issued policies to allow migrants to enroll in UEBMI or URBMI depending upon their employment status[11], and thus migrants may be enrolled in any of the three insurance programs. This may result in the variation in insurance benefits among migrants.

Secondly, the social health insurance programs are administrated and financed by local county or city government[12]. Each county or city designs its benefits package, which mainly covers health services delivered within the county/city and generally does not reimburse health services delivered outside of the county/city[10]. Even if

For peer review only - http://bmjopen.bmj.com/site/about/guidelines.xhtml

some counties/cities cover health services provided outside of the county/city, the lack of insurance portability across regions could create an additional barrier for insurance enrollees to receive the benefits. Migrants may enroll in health insurance at one place but move to another place and receive health services in this new place. They need to go back to their place of origin to get insurance reimbursement. To address the insurance reimbursement issue for migrants, China promoted immediate reimbursement for cross-province medical care in 2014[13]. However, the immediate reimbursement policy was only used for inpatient services, and immediate reimbursement for outpatient services started a pilot in a few regions in 2020[14]. Despite these policies, the separation between the location of health care use and health insurance coverage would still be barriers to equity benefits[15].

Previous benefit analysis among the general population showed that government subsidies for social health insurance were pro-rich for both inpatient and outpatient services from 2003 to 2013 in China, although inequity in benefit distribution had been narrowed[16-18]. Evidence from URBMI (between 2007 and 2011)[19] and the social health insurance (between 2014 to 2016)[20] revealed that the lower-income groups benefited less than the higher-income groups. The poorest groups within URBMI and NCMS were consistently more likely to forego hospitalization services recommended by doctors than their wealthier counterparts between 2008 to 2018[9]. With the fragmented feature and increased benefit disparities, social health insurance not only reinforced the existing rural-urban inequity but also generated a new inequity between urban residents and migrants who lived in urban areas[8]. In recent years, more literature documented barriers to preventing migrants from gaining access to health care[21-22] and health insurance had little influence on healthcare utilization among internal migrants[22-24]. Li's study further concluded that NCMS did not play a significant role in reducing out-of-pocket payments for elderly migrants between 2005 to 2014[25]. In addition to these disparities, there is a unique contributor to health inequity among migrants – the scope of migration – that they migrated to. However, no literature has focused on the benefit distribution of social health insurance by the scope of migration for the enormous migrant population in China.

Using the 2014 China National Internal Migrants Dynamic Monitoring Survey, we conducted a benefit analysis of social health insurance among a representative sample of migrants in China. This study was the first to assess the benefit distribution by the

For peer review only - http://bmjopen.bmj.com/site/about/guidelines.xhtml

geographic scope of migration and health insurance programs. We aimed to generate new evidence on China's continuously changing health insurance system and provide policy implications for other developing countries thriving to achieve universal health coverage under rapid urbanization.

METHODS

Data and Study Design

Data used for this analysis were from the 2014 China National Internal Migrant Dynamic Monitoring Survey. The survey was conducted by the National Health and Family Planning Commission of China in May 2014. This was a national cross-sectional survey representing 15-59-year-old internal migrants who have lived in a city of a new residence for more than one month but do not have a "Hukou" of the city (registered resident certificate).

In this survey, a stratified multi-stage random sampling method by Probability Proportional to Size (PPS) was employed, and the annual national data on internal migrants from each province in 2013 was considered as the primary sampling frame. A total of 348 cities from 32 provincial units in China were surveyed. Within each city, townships were randomly selected and followed by neighborhoods using the PPS. And then, in each neighborhood, 20 internal migrants were randomly selected to participate in the survey, finally reaching a total sample of 200,937 respondents. The face-to-face interview was conducted by trained interviewers using a structured questionnaire. This study was approved by the Ethics Committee of the School of Public health, Fudan University, China. Informed consent was sought from the study respondents.

Questionnaires included demographic information and family structures, socioeconomic status, migration characteristics, health insurance, health care services, and family planning services. In this study, we focused on internal migrants who used inpatient care in the city of new residence during the previous year of the survey and analyzed the benefits they received from their social health insurance. Thus, our sample comprised internal migrants who used inpatient care services in the city of a new residence and had social health insurance, with a sample size of 1165 in total.

Measurements

For peer review only - http://bmjopen.bmj.com/site/about/guidelines.xhtml

In this analysis, health insurance benefits were measured using three outcomes: the probability of receiving reimbursements from social health insurance, total amounts of reimbursement received, and the percentage of reimbursements of total health care expenditures (reimbursement ratio). In the survey, we identified the first outcome by a multiple-choice question, "where you received reimbursement for your last hospitalization this year," and answers included: allowance from NCMS, allowance from UEBMI, the employer, the NCMS office, the local health centers, the commercial insurance, allowance from the Family planning operation, the Family planning operation, and else. We recognized those who answered only "the commercial insurance" or "else" or both "the commercial insurance" and "else" as receiving no reimbursement from social health insurance. We identified the second outcome by the question "how much reimbursement did you receive from social health insurance." We identified the last outcome by the question for the second outcome and the question "how much did you cost in total."

Our primary predictors of interest were social health insurance programs and the geographic scope of migration. Social health insurance programs included UEBMI, URBMI, and NCMS. Since the social health insurance programs are administrated and financed by county or city, we hypothesized there are benefit disparities for migrants rooted in the separate administration of insurance programs. Therefore, our study categorized migrants according to their scope of migration, which can capture the degree of separate administration of insurance programs. The geographic scope of migration was categorized into three subgroups: migration across counties within a city, migration across cities within a province, and migration across provinces (Under China's administrative division, a county is smaller than a city).

Controlled variables included demographic characteristics, socioeconomic status, other migration characteristics, and the facility level for hospitalization. Demographic characteristics included gender, age, and marital status. Marital status was measured by a binary variable indicating whether the respondent was married or not married (e.g., widowed, divorced, or never married). Socioeconomic status was measured by educational attainment, monthly household income per capita, whether the respondent had a job, whether the respondent had rural "Hukou," and whether the respondent lived in urban areas. Educational attainment was categorized into four subgroups:

For peer review only - http://bmjopen.bmj.com/site/about/guidelines.xhtml

primary school and below, junior high school, senior high school, and college degree and above. “Hukou” represents the record in the residency registration system in China; people can be registered as having either a rural or urban “Hukou” at birth and cannot be easily changed throughout their lifetime[26]. Other migration characteristics were measured by reasons for and duration of migration. The reasons for migration included seeking jobs, family members following them to migrate, or other reasons. Migration duration was categorized into four groups: less than one year; one to five years; five to ten years; and ten years and above. Finally, the facility level for hospitalization included primary care facility, secondary hospital, tertiary hospital, and private hospital.

Statistical analysis

We first described the general characteristics of our study sample. Chi-square test and one-way variance analysis were used to compare the differences in the probability of receiving reimbursement and the amount and ratio of reimbursement received according to the geographic scope of migration.

Since there were many “zero observations” - patients who used inpatient care but received no reimbursement, we used the two-part model to estimate the benefits migrants received from the social health insurance, which can be expressed as follows:

$$\Pr\left[(\text{Reimburse amount})_i > 0 \,/\, (\text{Migration scope})_i\right] = \Phi[\beta * (\text{Migrantion scope})_i + \eta * X_i + \mathrm{e}_i] \quad (1)$$

$$log[(\text{Reimburse amount})_i / \,(\text{Reimburse amount})_i > 0, (\text{Migration scope})_i, \mathrm{X}_i] = \theta * (\text{Migration scope})_i + \gamma * X_i + \psi_i \quad (2)$$

$$(\text{Reimburse ratio})_i / \,(\text{Reimburse amount})_i > 0, (\text{Migration scope})_i, \mathrm{X}_i = \varepsilon * (\text{Migrantion scope})_i + \delta * X_i + \alpha_i \quad (3)$$

Where $(Reimburse\ amount)_i$ and $(Reimburse\ ratio)_i$ are the reimbursement amount and reimbursement ratio received by individual i. $(Migration\ scope)_i$ is a set of dummies representing migration scopes of individual i, and migrants who migrated across counties within a city are taken as the reference group. The parameters β, θ, and ε, the key coefficients of interest, identify the association between migration scope and the probability of receiving reimbursement and the

For peer review only - http://bmjopen.bmj.com/site/about/guidelines.xhtml

amount and ratio of reimbursement conditional on reimbursement received, respectively. X_i is a vector of control variables, including social health insurance coverage, demographic characteristics, socioeconomic status, other migration characteristics, and the facility level for hospitalization.

The above two-part model assumes that the benefits migrants received from the social health insurance is determined by two separate decision-making processes: equation (1), the 'participation equation,' captures the fundamental difference between the respondents who received reimbursements from social health insurance and those who did not; as the 'intensity equation,' equation (2) or equation (3) characterizes the determinants of the amount and the ratio of reimbursement received among those who received reimbursements. In equation (2) and equation (3), the amount and the ratio of the reimbursement fit the Gamma distribution, and the logarithm transformation was taken on the amount of the reimbursement to reduce the impact of extreme values. Following the previous studies [e.g., Jan pan, Sen Tian, Qin Zhou, and Wei Han (2016)[719], we estimated equation (1) with the Probit Model, and equation (2) and equation (3) with the Generalized Linear Model (GLM), respectively. Marginal effects with standard errors were reported.

All the analyses were conducted for the total sample, rural social insurance sample (the NCMS subsample), and urban social insurance sample (the URBMI & UEBMI subsample), respectively. All analyses were performed using STATA 12.0 (StataCorp LP, College Station, TX, USA).

Patient and public involvement

No patients were actively involved in setting the research question, outcome measures nor involved in the design of the study. Patients were not involved in interpretation or write up of the results, nor are there plans for the results to be disseminated to the patient community affected by this research.

Results

Characteristics of the study sample

Table 1 presents the descriptive statistics for our study sample. Of the 1165 respondents, 66.70% enrolled in NCMS, and 23.00% and 10.30% enrolled in UEBMI and URBMI, respectively. The average expenditures per inpatient stay were 10,366

For peer review only - http://bmjopen.bmj.com/site/about/guidelines.xhtml

Chinese Yuan (=1,567 US dollars), and there was a small difference (about 500 Yuan , P=0.643) in inpatient expenditure between the NCMS and URBMI & UEBMI subsample. 66.78% of respondents who used inpatient care received reimbursement from social health insurance, with 60.49% for NCMS enrollees and 79.38% for URBMI & UEBMI enrollees. Among the respondents who received reimbursement, the average amount and ratio of the reimbursement received were 5,506 Yuan (=832 US dollars) and 46.77%. The average amount and ratio of the reimbursement received for NCMS enrollees were much smaller than those for URBMI & UEBMI enrollees.

The average age and monthly household income per capita of the respondents were 38 years old and 2,256 Yuan. Less than half of the respondents were female. Most of them were married (89.27%), had an education level of high school or below (89.70%), had rural “Hukou” (87.81%), and owned a job (79.57%), and lived in urban areas (69.36%). Nearly half of the respondents migrated across provinces, while those who migrated across cities but within a province and those who migrated across counties but within a city were 28.76% and 25.41%, respectively. 84.21% of respondents migrated for better job opportunities, and 87.38% of respondents had lived in the city of a new residence for more than one year. Most respondents (80.51%) chose inpatient care at secondary and tertiary hospitals instead of primary care facilities.

Table 1 Characteristic of the study sample, n (%)

Variables	Total sample (N=1165)	NCMS subsample (N=777)	URBMI&UEBMI subsample (N=388)
Total expenditure per inpatient stay (Yuan) *	10366.05 (±17549.73)	10197.45 (±18374.85)	10704.99 (±15778.28)
Probability of receiving reimbursement	778(66.78)	470(60.49)	308(79.38)
Reimbursement amount received (Yuan) *	5506.16 (±9761.79)	5128.44 (±10838.10)	6049.14 (±7952.00)
Reimbursement ratio received (%) *	46.77(±19.91)	39.41(±17.86)	57.35(±17.87)
Social health insurance programs [a]			
NCMS	777(66.70)	-	-
URBMI	120(10.30)	-	120(30.93)
UEBMI	268(23.00)	-	268(69.07)
Age (Years) *	37.65(±9.75)	37.79(±9.80)	37.37(±9.65)
Female	532(45.67)	361(46.46)	171(44.07)
Married	1040(89.27)	705(90.73)	335(86.34)
Education attainment			
Primary school and below	279(23.95)	218(28.06)	61(15.72)
Junior high school	554(47.55)	416(53.54)	138(35.57)
Senior high school	212(18.20)	118(15.19)	94(24.23)

For peer review only - http://bmjopen.bmj.com/site/about/guidelines.xhtml

College and above	120(10.30)	25(3.22)	95(24.48)
Monthly household income per capita (Yuan) *	2256.43(±2092.85)	1999.55(±1687.47)	2770.86(±2658.26)
Having any job	927(79.57)	610(78.51)	317(81.70)
Rural Hukou	1023(87.81)	777(100.00)	267(68.81)
Living in an urban area	808(69.36)	502(64.61)	306(78.87)
Geographic scope of migration			
Across counties within a city	296(25.41)	216(27.80)	80(20.62)
Across cities within a province	335(28.76)	214(27.54)	121(31.19)
Across provinces	534(45.84)	347(44.66)	187(48.20)
Reasons for migration			
Seeking jobs	981(84.21)	642(82.63)	339(87.37)
Family members following migrants	146(12.53)	115(14.80)	31(7.99)
Other reasons	38(3.26)	20(2.57)	18(4.64)
Migration duration (Years)			
0~1	147(12.62)	117(15.06)	30(7.73)
1~5	484(41.55)	305(39.25)	179(46.13)
5~10	272(23.35)	181(23.29)	91(23.45)
10 +	262(22.49)	174(22.39)	88(22.68)
Facility level of hospitalization			
Primary care facility	131(11.24)	90(11.58)	41(10.57)
Secondary hospital	400(34.33)	282(36.29)	118(30.41)
Tertiary hospital	538(46.18)	340(43.76)	198(51.03)
Private hospital	96(8.24)	65(8.37)	31(7.99)

Note: * mean (± Standard Deviation).

a. NCMS: New Rural Cooperative Medical Scheme; URBMI: Urban Resident Basic Medical Insurance; UEBMI: Urban Employee Basic Medical Insurance.

We compared the characteristics of the study sample by the scope of migration (Appendix Table 1). The demographic and socio-economic characteristics of migrants were similar across three scopes of migration subgroups, including age, sex, marriage status, education, job status, Hukou status, reasons for migration, migration duration, and health insurance programs they enrolled in. The Only exception was income and living areas, and migrants across provinces had more income and were less likely to live in urban areas than other groups.

Table 2 summarizes the total expenditures per inpatient stay, and the probability of benefiting from the social health insurance. It also presents that among the benefit recipients, the amount and ratio of reimbursement received according to the

For peer review only

For peer review only - http://bmjopen.bmj.com/site/about/guidelines.xhtml

geographic scope of migration. The univariate analysis showed that the broader the migration scope, the lower the probability that migrants would receive reimbursements; but among those who received reimbursements, those who migrated across cities or across provinces received the larger amounts of reimbursement than those who migrated within a city. There was no significant difference in total expenditure and reimbursement ratio by the geographic scope of migration.

Table 2 Benefit distribution of social health insurance by geographic scope of migration

Variables	Across counties within a city	Across cities within a province	Across provinces	P
Total expenditure per inpatient stay (Yuan)	10326.81 (14079.35)	14458.96 (27139.10)	12771.68 (20428.78)	0.354
Probability of receiving reimbursement (%)	81.76 (38.69)	69.85 (45.96)	56.55 (49.62)	<0.001
Reimbursement amount received (Yuan)	4402.12 (6541.01)	6706.32 (13681.56)	5495.89 (8105.80)	0.056
Reimbursement ratio received (%)	44.75 (18.17)	47.76 (20.51)	47.72 (20.79)	0.195

Note: Standard Deviations are reported in parentheses.

Association between insurance programs, migration scope, and social health insurance benefits

Table 3 reports the association between insurance programs, migration scope, other factors, and social health insurance benefits, estimated from a two-part model. Compared with NCMS enrollees, URBMI or UEBMI enrollees were more likely to receive reimbursement, and among the benefit recipients, urban insurance enrollees received a larger reimbursement amount and ratio. The probability of receiving reimbursement for UEBMI enrollees was 37.5% ($P<0.01$) higher than that for the NCMS enrollees. Among insurance benefit recipients, UEBMI enrollees received 42.8% ($P<0.01$) more reimbursement amount and a 20.1% ($P<0.01$) higher reimbursement ratio than NCMS enrollees.

According to the association between insurance benefits and migration scope, the geographic scope of migration significantly reduced the probability of receiving reimbursement and the reimbursement ratio but increased the reimbursement amounts they received. Specifically, the probability of receiving reimbursement for those who migrated across cities and provinces was 14.7% and 26.0%, respectively, lower than

For peer review only - http://bmjopen.bmj.com/site/about/guidelines.xhtml

those who migrated within a city (P<0.01). However, they received 33.4% and 27.2% higher reimbursement amounts than those who migrated within a city (P<0.01). And those who migrated across provinces had the lowest reimbursement ratio (P<0.10).

In addition, there was no significant difference in insurance benefits by age, gender, marriage status, education, Hukou status, and migration duration. Income had no significant influence on the probability of receiving reimbursement and reimbursement ratio but significantly increased the reimbursement amount. Having jobs significantly decreased the probability and amount of receiving reimbursement, whereas living in urban areas significantly increased the probability of receiving reimbursement by 6.3% than living in suburban areas. Compared with migration for seeking jobs, family members following migrants significantly increased the reimbursement amount and ratio. The higher the level of health care facility, the greater probability and amount of receiving reimbursement, but the lower the reimbursement ratio.

For peer review only

Table 3 Association between insurance programs, migration scope, other factors, and social health insurance benefits

Variables	Probit Model		Generalized Linear Model			
	Probability of getting reimbursements		Reimbursement amount		Reimbursement ratio	
	Coefficient	S.E.	Coefficient	S.E.	Coefficient	S.E.
Social health insurance (referred to NCMS)						
URBMI	0.021	0.049	0.222	0.136	0.147***	0.031
UEBMI	0.375***	0.044	0.428***	0.094	0.201***	0.023
Age (years)	0.010	0.012	0.026	0.030	0.006	0.006
Age^2 (years)	0.000	0.000	0.000	0.000	0.000	0.000
Female	0.024	0.031	-0.004	0.078	0.018	0.016
Married	0.063	0.051	-0.037	0.146	0.010	0.032
Education attainment (referred to primary school and below)						
Junior high school	0.039	0.038	0.108	0.099	0.014	0.020
Senior high school	0.081	0.050	0.014	0.125	0.027	0.026
College and above	-0.038	0.068	-0.015	0.173	0.032	0.039
Monthly household income per capita (referred to first quintile)						
2nd quintile	0.062	0.048	0.328***	0.124	-0.029	0.026
3rd quintile	-0.033	0.051	0.014	0.139	-0.041	0.028
4th quintile	0.047	0.049	0.179	0.124	-0.031	0.026
5th quintile	0.029	0.053	0.336**	0.137	-0.023	0.030
Having any job	-0.070*	0.042	-0.247**	0.109	0.010	0.022
Rural hukou	0.088	0.053	-0.106	0.126	0.000	0.031

For peer review only - http://bmjopen.bmj.com/site/about/guidelines.xhtml

Living in an urban area	0.063**	0.032	-0.012	0.088	0.004	0.017
Geographic scope of migration (referred to migration across counties within a city)						
Across cities within a province	-0.147***	0.041	0.334***	0.093	-0.019	0.019
Across provinces	-0.260***	0.038	0.272***	0.093	-0.032*	0.019
Reasons for migration (referred to Seeking jobs)						
Family members following migrants	-0.007	0.050	0.553***	0.137	0.047*	0.027
other reasons	-0.025	0.087	-0.224	0.207	0.024	0.048
Migration duration (referred to 0~1)						
1~5	-0.014	0.045	0.078	0.125	-0.032	0.026
5~10	0.017	0.051	0.162	0.135	-0.022	0.028
10 +	-0.011	0.052	0.085	0.140	-0.040	0.029
Facility level of hospitalization (referred to primary care facility)						
Secondary hospital	-0.031	0.048	0.603***	0.139	-0.046	0.032
Tertiary hospital	0.083*	0.048	1.065***	0.135	-0.083***	0.031
Private hospital	-0.156**	0.063	0.092	0.188	-0.027	0.043
Observations	1165		663		663	

Note: ***, ** and * denote statistical significance at 1, 5 and 10% level, respectively. S.E.: Standard Error. NCMS: New Rural Cooperative Medical Scheme; URBMI: Urban Resident Basic Medical Insurance; UEBMI: Urban Employee Basic Medical Insurance.

Considering the differences in reimbursement policy between NCMS and urban health insurance, we further conducted the above regressions among the subsamples of NCMS enrollees and URBMI & UEMBI enrollees (Table 4). The relationships between migration scope and the probability of receiving reimbursement and the reimbursement amount did not change, but it differed with the reimbursement ratio. Those who migrated more broadly had a significantly lower reimbursement ratio among NCMS enrollees but had a significantly higher ratio among URBMI & UEMBI enrollees.

Table 4 Association between migration scope and benefit of social health insurance programs

Variables	Probit Model		Generalized Linear Model			
	Probability of getting reimbursements		Reimbursement amount		Reimbursement ratio	
	Coefficient	S.E.	Coefficient	S.E.	Coefficient	S.E.
**NCMS subsample**						
Geographic scope of migration (referred to migration across counties within a city)						

For peer review only - http://bmjopen.bmj.com/site/about/guidelines.xhtml

Across cities within a province	-0.139***	0.052	0.410***	0.120	-0.044*	0.023
Across provinces	-0.333***	0.048	0.282**	0.118	-0.055**	0.024
**URBMI & UEBMI subsample**						
Geographic scope of migration (referred to migration across counties within a city)						
Across cities within a province	-0.135**	0.061	0.339**	0.154	0.073**	0.033
Across provinces	-0.118*	0.061	0.285*	0.152	0.039	0.033

Note: All models included confounding factors in Table 3. ***, **, and * denote statistical significance at 1%, 5%, and 10% levels.

S.E.: Standard Error. NCMS: New Rural Cooperative Medical Scheme; URBMI: Urban Resident Basic Medical Insurance; UEBMI: Urban Employee Basic Medical Insurance.

Reasons for not receiving reimbursement from social health insurance

We further investigated the reasons why migrants did not receive reimbursement from their social health insurance. Figure 1 showed that the need or plan to go back to hometown to get reimbursement was the main reason for not getting reimbursement, accounting for 66.5%, followed by a lack of knowledge about the reimbursement process (16.7%) and the policy coverage issues (10.6%). Figure 2 further compares the proportion of not receiving reimbursement due to the need or plan to go back to their hometown by migration scope. The broader the migration scope, the higher the likelihood that migrants did not receive reimbursement because they must get the reimbursement later from their hometowns.

Discussion

Using data from the 2014 China National Internal Migrants Dynamic Monitoring Survey, this study documented social health insurance benefit distribution among internal migrants. Among migrants who utilized inpatient care in 2013, only 67% received reimbursements from their social health insurance, and the reimbursement amount only accounted for 47% of the total expenditure per inpatient stay. There were large disparities in health insurance programs and migration scope. The broader the migration scope, the lower the probability of receiving reimbursement and the reimbursement ratio, but the higher the reimbursement amount.

For peer review only

For peer review only - http://bmjopen.bmj.com/site/about/guidelines.xhtml

We found that the probability of receiving reimbursement and the reimbursement ratio for migrants were far smaller than those for the general population. Only 60% of migrants who were NCMS enrollees received reimbursement from NCMS, which was 30% lower than that for general NCMS enrollees (91.1% in 2013), whereas the reimbursement ratio among this group of migrants was 10% lower than that among general NCMS enrollees (39.4% vs. 50.1% in 2013)[27]. For URBMI & UEBMI enrollees, the probability of receiving reimbursement among migrants was about 10% lower than that among the general population (79.4% vs. 95.3% for UEBMI and 88.7% for URBMI enrollees), although a small difference existed in the reimbursement ratio between the two groups (around 60%)[27]. A previous study also pointed out that migrants only partially benefited from health insurance coverage[24]

The results should be understood in the China-specific context. There were several challenges for migrants to get insurance benefits under the current insurance policy design. In China, migrants face more challenges in getting insurance reimbursement than non-migrants. There have been two common approaches to reimburse health care services (immediate reimbursement and later reimbursement)[28]. Immediate reimbursement means that the insured patient gets reimbursement immediately for the treatment and only pays out-of-pocket for the copay or coinsurance rate, whereas later reimbursement means that the insured patient pays the total expenditures out-of-pocket up-front and gets reimbursement later from their health insurance[28]. Local residents usually get reimbursement immediately, but migrants, generally, receive reimbursement later as they must travel back to their hometown where they enroll in social health insurance to receive reimbursement. Our study showed that up to 22% of migrants reported that they did not get reimbursement because they needed to get reimbursement from their hometowns. In addition, research has shown that many services were not reimbursed, and the reimbursement process was much more complex for migrants than for local residents[29]. One study showed that more migrants were treated in a hospital outside of the NCMS designated network than local residents, and thus their healthcare use was less likely to be covered by NCMS[29].

Our findings also showed inequity in insurance benefits among migrants by migration scope. Although the scope of migration was associated with a larger reimbursement amount per inpatient stay, it was significantly associated with a lower probability of receiving reimbursement and a lower reimbursement ratio. In China, all three social

For peer review only - http://bmjopen.bmj.com/site/about/guidelines.xhtml

health insurance programs were administered, financed, and operated by local county or city governments. Each county or city designed its own benefits packages and made the benefit localized[10,30], limiting individual coverage choices outside the local region. This poses a challenge for internal migrants who typically use health care in the city of new residence but may enroll in health insurance in their hometown according to their residence ("Hukou") status. The separation between where health care is received and where health insurance is administrated provided an additional hurdle for the internal migrants to receive benefits from their social health insurance. It became even more difficult when they lived far away from their hometowns. The localized administration of social health insurance and the later reimbursement approach contributed jointly to the inequity in benefit coverage for migrants[31-32]. To resolve this inequity, China has made efforts to improve the portability of social health insurance through policy development and the building of a national health insurance information platform. However, till now, the information platform among different provinces has not been fully interconnected[33]. Moreover, these efforts mainly focus on insurance reimbursement for cross-province inpatient care rather than outpatient care.

Another important finding of this study was that migrants who got reimbursement received larger reimbursement amounts if they migrated more broadly. Compared to migration within a city, migration across cities or across provinces was significantly associated with a 30% higher reimbursement amount per inpatient stay. This is mainly attributed to higher health care costs in larger cities and more affluent regions where migrants who migrated more broadly like to locate. However, these reimbursements were mainly paid by health insurance funds in smaller counties/cities which were less affluent. This may pose financial difficulties for local governments in those out-migration regions as if the current barriers to reimbursement were to be removed, and there would have been more significant amounts of insurance funds flowing into the health care system in more prosperous in-migration regions, which would worsen the already skewed regional inequity in economic development and health. Therefore, the inverse subsidies of health insurance funds from the less-developed out-migration regions to the highly-developed in-migration regions became an ongoing challenge and policy dilemma for countries like China that experience a mass domestic migration. Strategies that control health expenditures inflation and allow the central

For peer review only - http://bmjopen.bmj.com/site/about/guidelines.xhtml

government to redistribute welfare funding to less-developed regions may be warranted to address the financial difficulties faced by out-migration regions[6].

In addition to the above inequity related to migration, there were two other types of inequity in benefits due to the fragmented social insurance system and income inequity. Although China has almost achieved universal insurance coverage through social health insurance expansion[6], migrants who enrolled in UEBMI and URBMI benefited more than those enrolled in NCMS[1034]. Several national studies found that among the general population, UEBMI enrollees had a higher benefit level than those covered by URBMI or NCMS[35-36]. Even for the same condition - tuberculosis inpatient care- the reimbursement rate was the highest for UEBMI enrollees, followed by URBMI, and NCMS enrollees in 2012[34]. Income inequity also explains some of the benefit inequities. Among the benefit recipients, we found that migrants with higher income received more significant reimbursement amounts, which was consistent with prior studies among the general population[161937]. On average, the higher-income group tended to have higher inpatient expenditure, and it was not surprising that they also received greater reimbursement amount[38]

This study contained some limitations. First, the outcomes related to reimbursement were self-reported, leading to measurement bias. Future research will use health insurance claims data to minimize this bias. Second, health status may influence health care utilization as well as whether a person chooses to migrate. Studies have detected the healthy migrant effect for internal migrants in China, showing that healthier people were more likely to migrate and to move farther away from home[39]. Unfortunately, there was no measurement of the health status of migrants in this dataset. Third, we could not accurately distinguish the administrative site of social health insurance, which may affect the insurance benefit for internal migrants. To reduce this bias, we conducted the analysis separately on two subsamples of NCMS enrollees and UEBMI & URBMI enrollees.

Conclusions

This study has important policy implications not only for China but also for other developing countries that experience rapid urbanization and internal migration. In some developing countries (e.g., Mexico), health insurance programs are

For peer review only

For peer review only - http://bmjopen.bmj.com/site/about/guidelines.xhtml

administrated locally and separated across regions[40-41], hindering the insurance benefits when people migrate across regions. Internal migrants often move beyond the administrated region for work and then would face the same problem as the Chinese when seeking health services.

In China, the broader the migration scope, the lower the probability of receiving reimbursements from the social insurance and the reimbursement ratio; but among those who received reimbursements, the broader the migration scope, the larger amounts they were reimbursed for health care use. This unequal benefit distribution may be attributed to the fragmented insurance design that relies on localized administration and patients paying services up-front and requesting reimbursement later. To improve the equity in social insurance benefits, China has been promoting the portability of social health insurance and immediate reimbursement for inpatient care utilized across regions and a more integrated health insurance system. Efforts should also be made to control inflation of health care expenditures and prevent inverse government subsidies from out-migration regions to in-migration regions.

For peer review only - http://bmjopen.bmj.com/site/about/guidelines.xhtml

Contribution Statement

ZH designed the study and drafted the manuscript. HW conducted the literature review, analyzed the data, and contributed to the manuscript writing. DL and DZ revised the manuscript. All authors read and approved the final manuscript being submitted.

Acknowledgments

We thank the National Health and Family Planning Commission of China for providing the data.

Competing Interests

None.

Funding

This work was supported by the National Natural Science Foundation of China (No. 71874034) and the National Key R&D Program of China (No. 2018YFC1312600 and 2018YFC1312604).

Data availability statement

The data used in this paper were provided by the National Health and Family Planning Commission of China. We signed a legally binding agreement with the Commission that we would not share the original data with any third parties. Although researchers who are interested can apply to get access to the data at: http://www.moh.gov.cn/ldrks/s7848s/201510/8d4c69223d4c49ca871fdd6fdc57aba5.shtml and Email: ldrkzxsj@163.com, it is up to the National Health and Family Planning Commission to make the final decision.

For peer review only

For peer review only - http://bmjopen.bmj.com/site/about/guidelines.xhtml

References

1 P Gong, S Liang, EJ Carlton, et al. Urbanisation and health in China. *The Lancet* 2012;9818(379):843-52.

2 National Bureau of Statistics. Bulletin of the Seventh National Population Census (No. 7). In, 2021.

3 JX Fan, M Wen, L Jin, G Wang. Disparities in Healthcare Utilization in China: Do Gender and Migration Status Matter? *J Fam Econ Iss* 2013;34(1):52-63.

4 S Wang, A Liu, W Guo. Public and Commercial Medical Insurance Enrollment Rates of Rural-to-Urban Migrants in China. *Frontiers in Public Health* 2021;9.

5 Y Jin, Z Hou, D Zhang. Determinants of Health Insurance Coverage among People Aged 45 and over in China: Who Buys Public, Private and Multiple Insurance. *Plos One* 2016;11(8):e161774.

6 Q Meng, H Fang, X Liu, B Yuan, J Xu. Consolidating the social health insurance schemes in China: towards an equitable and efficient health system. *Lancet* 2015;386(10002):1484-92.

7 Z Hou, E Van de Poel, E Van Doorslaer, B Yu, Q Meng. EFFECTS OF NCMS ON ACCESS TO CARE AND FINANCIAL PROTECTION IN CHINA. *Health Econ* 2014;23(8):917-34.

8 X Huang. Expansion of Chinese Social Health Insurance: who gets what, when and how? *J Contemp China* 2014;23(89):923-51.

9 X Yan, Y Liu, M Cai, et al. Trends in disparities in healthcare utilisation between and within health insurances in China between 2008 and 2018: a repeated cross-sectional study. *Int J Equity Health* 2022;21(1).

10 Q Wang, D Zhang, Z Hou. Insurance coverage and socioeconomic differences in patient choice between private and public health care providers in China. *Soc Sci Med* 2016;170:124-32.

11 Ministry of Human Resources and Social Security of the People's Republic of China. Interim Measures for the Transferability of Basic Medical Insurance Account among Internal Migrants. In, 2009.

12 M Ramesh, X Wu, AJ He. Health governance and healthcare reforms in China. *Health Policy Plann* 2014;29(6):663-72.

13 General Office of the State Council. Notice on key missions for deepeng health system reform in 2014. In, 2014.

14 National Healthcare Security Administration. Notice on Promoting the Pilot Program of Cross-Provincial Direct Settlement of Outpatient Expenses. In, 2020.

15 S Chen, Y Chen, Z Feng, et al. Barriers of effective health insurance coverage for rural-to-urban migrant workers in China: a systematic review and policy gap analysis. *Bmc Public Health* 2020;20(1).

16 M Chen, G Fang, L Wang, et al. Who Benefits from Government Healthcare Subsidies? An Assessment of the Equity of Healthcare Benefits Distribution in China. *Plos One* 2015;10(3):e119840.

17 M Chen, AJ Palmer, L Si. Assessing equity in benefit distribution of government health subsidy in 2012 across East China: benefit incidence analysis. *Int J Equity Health* 2016;15(1).

18 L Si, M Chen, AJ Palmer. Has equity in government subsidy on healthcare improved in China? Evidence from the China’ s National Health Services Survey. *Int J Equity Health* 2017;16(1).

19 J Pan, S Tian, Q Zhou, W Han. Benefit distribution of social health insurance: evidence from china’ s urban resident basic medical insurance. *Health Policy Plann* 2016;31(7):853-59.

20 L Diao, Y Liu. Inequity under equality: research on the benefits equity of Chinese basic medical

For peer review only

For peer review only - http://bmjopen.bmj.com/site/about/guidelines.xhtml

insurance. *Bmc Health Serv Res* 2020;20(1).

21 X Ke, L Zhang, Z Li, W Tang. Inequality in health service utilization among migrant and local children: a cross-sectional survey of children aged 0－14 years in Shenzhen, China. *Bmc Public Health* 2020;20(1).

22 F Zhang, X Shi, Y Zhou. The Impact of Health Insurance on Healthcare Utilization by Migrant Workers in China. *Int J Env Res Pub He* 2020;17(6):1852.

23 H Wang, D Zhang, Z Hou, F Yan, Z Hou. Association between social health insurance and choice of hospitals among internal migrants in China: a national cross-sectional study. *Bmj Open* 2018;8(2):e18440.

24 X Qin, J Pan, GG Liu. Does participating in health insurance benefit the migrant workers in China? An empirical investigation. *China Econ Rev* 2014;30:263-78.

25 J Li, Y Huang, S Nicholas, J Wang. China’s New Cooperative Medical Scheme’s Impact on the Medical Expenses of Elderly Rural Migrants. *Int J Env Res Pub He* 2019;16(24):4953.

26 Z Zhang, DJ Treiman. Social origins, hukou conversion, and the wellbeing of urban residents in contemporary China. *Soc Sci Res* 2013;42(1):71-89.

27 National Health Commission of the People's Republic of China. An Analysis Report of National Health Services Survey in China，2013. Beijing: Peking Union Medical College Press 2015.

28 H Zhong. Effect of patient reimbursement method on health-care utilization: evidence from China. *Health Econ* 2011;20(11):1312-29.

29 P Qiu, Y Yang, J Zhang, X Ma. Rural-to-urban migration and its implication for new cooperative medical scheme coverage and utilization in China. *Bmc Public Health* 2011;11:520.

30 Y Zhao, B Kang, Y Liu, et al. Health insurance coverage and its impact on medical cost: observations from the floating population in China. *Plos One* 2014;9(11):e111555.

31 Q Gao, S Yang, S Li. Labor contracts and social insurance participation among migrant workers in China. *China Econ Rev* 2012;23(4):1195-205.

32 KKF Lam, JM Johnston. Health insurance and healthcare utilisation for Shenzhen residents: a tale of registrants and migrants? 2012;12(1):868.

33 Y Chen. Study On The Optimization of Long-Distance Medical Insurance of Public Hospitals In Shanghai: Based on Holistic Governance Theory. *Shanghai Jiao Tong University*, 2020.(Chinese)

34 Y Pan, S Chen, M Chen, et al. Disparity in reimbursement for tuberculosis care among different health insurance schemes: evidence from three counties in central China. *Infect Dis Poverty* 2016;5:7.

35 L X,J WY. Empirical Study on the Benefit Equity of Essential Health Insurance System in China. *medicine and Society* 2010;23(11):45-47.(Chinese)

36 W Dong, AB Zwi, R Bai, C Shen, J Gao. Benefits Associated with China’s Social Health Insurance Schemes: Trend Analysis and Associated Factors Since Health Reform. *Int J Env Res Pub He* 2021;18(11):5672.

37 S Yuan, C Rehnberg, X Sun, X Liu, Q Meng. Income related inequalities in New Cooperative Medical Scheme: a five-year empirical study of Junan County in China. 2014;13(1):38.

38 Z Zhou, J Gao, A Fox, et al. Measuring the equity of inpatient utilization in Chinese rural areas. *Bmc Health Serv Res* 2011;11:201.

39 Y Lu, L Qin. Healthy migrant and salmon bias hypotheses: a study of health and internal migration in China. *Soc Sci Med* 2014;102:41-48.

40 XY Li, Y Zhu, ZL Wu. Comparison of health financing mechanisms under the perspective of

For peer review only

universal health coverage in developing countries. *Jiangsu J Prev Med* 2017;28(03):254-58.(Chinese)

41 FM Knaul, E González-Pier, O Gómez-Dantés, et al. The quest for universal health coverage: achieving social protection for all in Mexico. *Lancet* 2012;380(9849):1259-79.

For peer review only

For peer review only - http://bmjopen.bmj.com/site/about/guidelines.xhtml

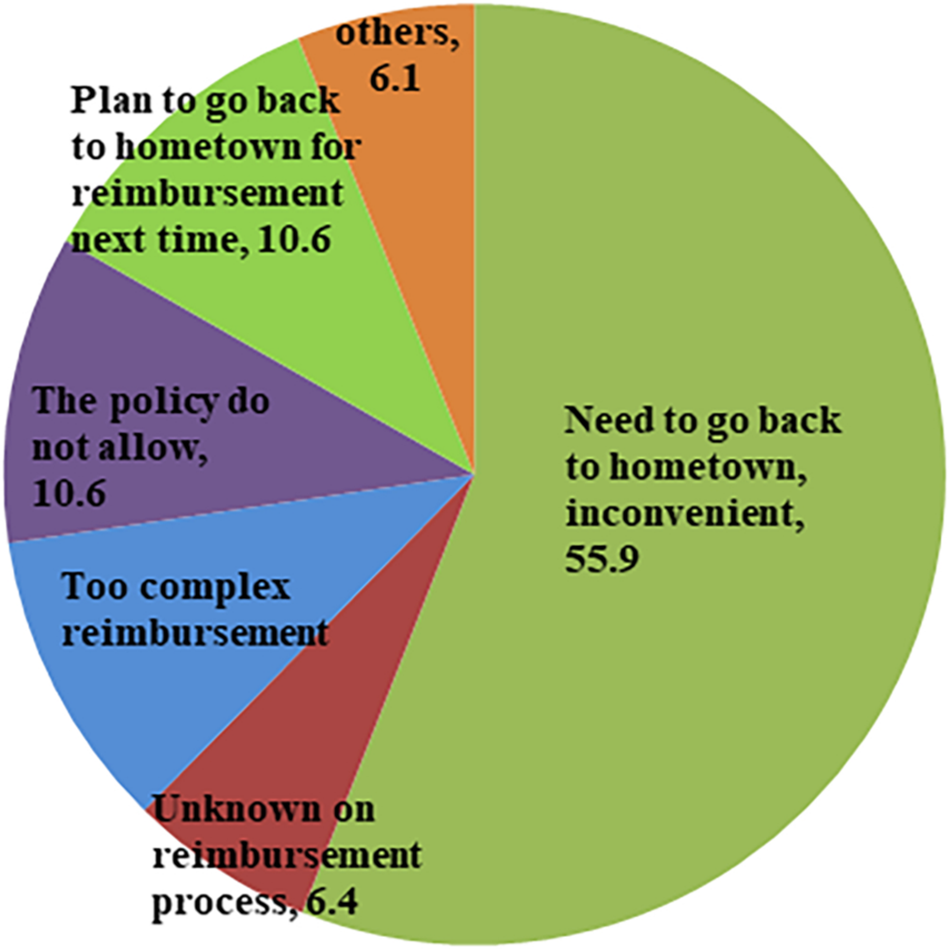

Figure 1. Reasons not getting reimbursement from social health insurance

228x242mm (118 x 118 DPI)

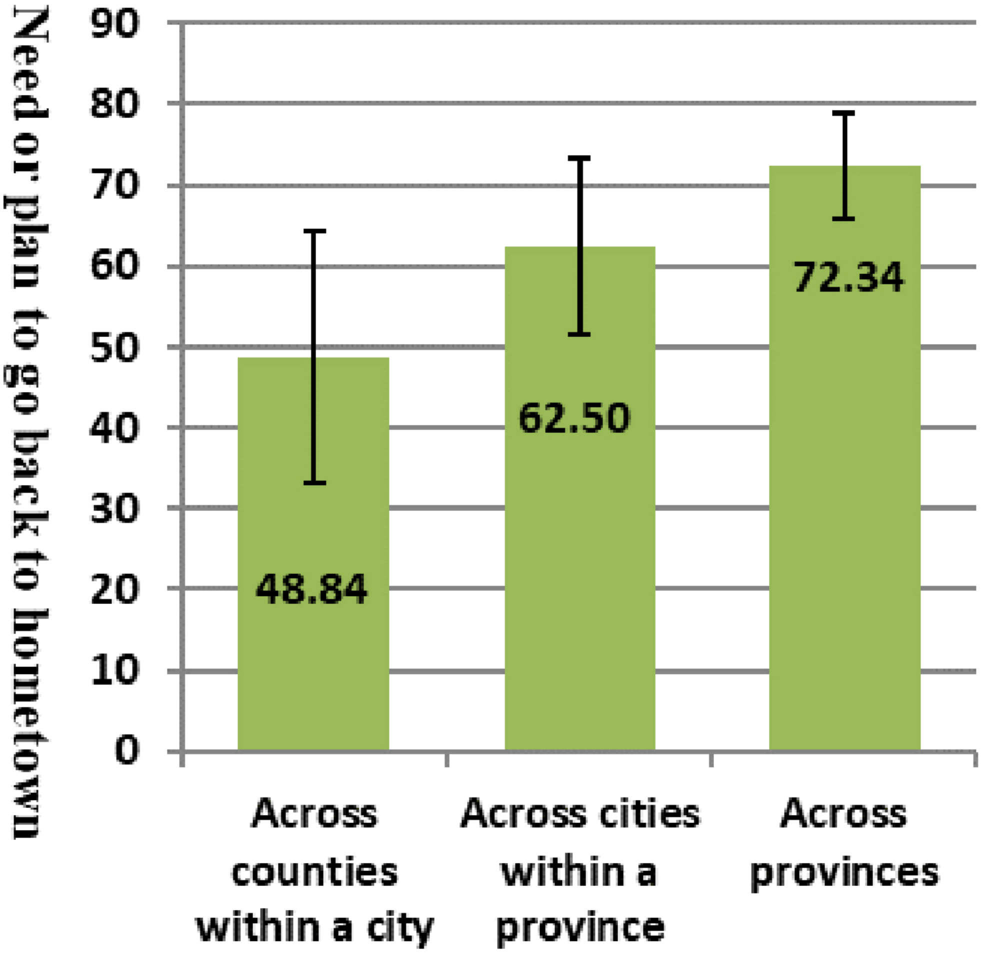

Figure 2. Proportion of not getting reimbursement due to the need or plan to go back to hometown for reimbursement, by geographic scope of migration

228x235mm (118 x 118 DPI)

Appendix Table 1 Characteristics of the study sample by migration scope

	Across counties within a city	Across cities within a province	Across provinces	F/χ2	P value
Age (Years) *	38.41(±10.35)	37.99(±9.62)	37.01 (±9.47)	2.248	0.106
Female	137(46.28%)	154(46.0%)	241(45.13%)	0.12	0.942
Married	262(88.51%)	298(88.96%)	480(89.89%)	0.424	0.809
Education attainment				6.979	0.323
Primary school and below	72(24.32%)	78(23.28%)	129(24.16%)		
Junior high school	137(46.28%)	154(45.97%)	263(49.25%)		
Senior high school	64(21.62%)	62(18.51%)	86(16.10%)		
College and above	23(7.77%)	41(12.24%)	56(10.49%)		
Monthly household income per capita (Yuan)*	1830.88(±1272.07)	1968.94(±1589.62)	2672.67(±2600.04)	20.506	0.000
Having any job	226(76.35%)	264(78.81%)	437(81.84%)	3.692	0.158
Rural Hukou	268(90.54%)	293(87.46%)	462(86.52%)	2.934	0.231
Living in an urban area	237(80.07%)	241(71.94%)	330(61.80%)	31.386	0.000
Reasons for migration				8.13	0.087
Seeking jobs	242(81.76%)	273(81.49%)	466(87.27%)		
Family members following migrants	44(14.86%)	51(15.22%)	51(9.55%)		
Other reasons	10(3.38%)	11(3.28%)	17(3.18%)		
Migration duration (Years)				10.475	0.106
0~1	43(14.53%)	32(9.55%)	72(13.48%)		
1~5	135(45.61%)	135(40.30%)	214(40.07)		
5~10	65(21.96%)	80(23.88%)	127(23.78%)		
10 +	53(17.91%)	88(26.27%)	121(22.66%)		
Social health insurance programs				8.376	0.079
NCMS	216(72.97%)	214(63.88%)	347(64.98%)		
URBMI	28(9.50%)	39(11.64%)	53(9.93%)		
UEBMI	52(17.57%)	82(24.48%)	134(25.09%)		

Note: * mean (± Standard Deviation).

For peer review only

BMJ Open

How does domestic migration pose a challenge in achieving equitable social health insurance benefits in China? a National Cross-Sectional Study

Journal:	*BMJ Open*
Manuscript ID	bmjopen-2021-060551.R2
Article Type:	Original research
Date Submitted by the Author:	22-Jul-2022
Complete List of Authors:	Wang, Haiqin; The International Peace Maternity & Child Health Hospital of China Welfare Institute, Administrative Office; Fudan University, School of Public Health, NHC Key Laboratory of Health Technology Assessment<br>Liang, Di; Fudan University, School of Public Health, NHC Key Laboratory of Health Technology Assessment<br>Zhang, Donglan; New York University Long Island School of Medicine, Division of Health Services Research, Department of Foundations of Medicine<br>Hou, Zhiyuan; Fudan University, School of Public Health, NHC Key Laboratory of Health Technology Assessment
**Primary Subject Heading**:	Health policy
Secondary Subject Heading:	Health policy, Health economics
Keywords:	Health policy < HEALTH SERVICES ADMINISTRATION & MANAGEMENT, HEALTH SERVICES ADMINISTRATION & MANAGEMENT, Health economics < HEALTH SERVICES ADMINISTRATION & MANAGEMENT

SCHOLARONE™
Manuscripts

I, the Submitting Author has the right to grant and does grant on behalf of all authors of the Work (as defined in the below author licence), an exclusive licence and/or a non-exclusive licence for contributions from authors who are: i) UK Crown employees; ii) where BMJ has agreed a CC-BY licence shall apply, and/or iii) in accordance with the terms applicable for US Federal Government officers or employees acting as part of their official duties; on a worldwide, perpetual, irrevocable, royalty-free basis to BMJ Publishing Group Ltd ("BMJ") its licensees and where the relevant Journal is co-owned by BMJ to the co-owners of the Journal, to publish the Work in this journal and any other BMJ products and to exploit all rights, as set out in our licence.

The Submitting Author accepts and understands that any supply made under these terms is made by BMJ to the Submitting Author unless you are acting as an employee on behalf of your employer or a postgraduate student of an affiliated institution which is paying any applicable article publishing charge ("APC") for Open Access articles. Where the Submitting Author wishes to make the Work available on an Open Access basis (and intends to pay the relevant APC), the terms of reuse of such Open Access shall be governed by a Creative Commons licence – details of these licences and which Creative Commons licence will apply to this Work are set out in our licence referred to above.

Other than as permitted in any relevant BMJ Author's Self Archiving Policies, I confirm this Work has not been accepted for publication elsewhere, is not being considered for publication elsewhere and does not duplicate material already published. I confirm all authors consent to publication of this Work and authorise the granting of this licence.

For peer review only

How does domestic migration pose a challenge in achieving equitable social health insurance benefits in China? a National Cross-Sectional Study

Haiqin Wang[a, b], Di Liang[b], Donglan Zhang[c], Zhiyuan Hou[b]*

a. Administrative Office, The International Peace Maternity & Child Health Hospital of China Welfare Institute, 910 Heng Shan Road, Shanghai 200030, China

b. School of Public Health, NHC Key Laboratory of Health Technology Assessment, Fudan University, 138 Yi Xue Yuan Road, Shanghai 200032, China

c. Division of Health Services Research, Department of Foundations of Medicine, New York University Long Island School of Medicine, 101 Mineola Blvd, Mineola, NY, 11501, USA

*Corresponding to Zhiyuan Hou, Ph.D., Associate professor,

School of Public Health, NHC Key Laboratory of Health Technology Assessment, Fudan University

Address: 138 Yi Xue Yuan Road, Shanghai 200032, China

Email: zyhou@fudan.edu.cn

Word count: 4196

For peer review only - http://bmjopen.bmj.com/site/about/guidelines.xhtml

Abstract

Objectives: To evaluate the benefit distribution of social health insurance among internal migrants in China.

Design: A national cross-sectional survey.

Setting: 348 cities from 32 provincial units in China.

Participants: 1165 internal migrants who used inpatient care services in the city of a new residence and had social health insurance.

Primary and secondary outcome measures: The probability of receiving reimbursements from social health insurance, the amounts and ratio of reimbursement received.

Results: Among migrants who utilized inpatient care in 2013, only 67% received reimbursements from social health insurance, and the reimbursement amount only accounted for 47% of the inpatient care expenditure. The broader the geographic scope of migration, the lower the probability of receiving reimbursement and the reimbursement ratio, but the higher the reimbursement amount. Specifically, the probability of receiving reimbursements for those who migrated across cities and provinces was 14.7% and 26.0%, respectively, significantly lower than those who migrated within a city (P<0.01). However, they received 33.4% and 27.2% higher reimbursement amounts than those who migrated within a city (P<0.01). And those who migrated across provinces had the lowest reimbursement ratio although not reaching significance level.

Conclusions: The unequal benefit distribution may be attributed to the fragmented health insurance design that relies on localized administration and patients paying services up-front and requesting reimbursement later. To improve the equity in social insurance benefits, China has been promoting the portability of social health insurance, immediate reimbursement for inpatient care utilized across regions, and a more integrated health insurance system. Efforts should also be made to control inflation of health care expenditures and prevent inverse government subsidies from out-migration regions to in-migration regions. This study has policy implications for China and other developing countries that experience rapid urbanization and internal

For peer review only - http://bmjopen.bmj.com/site/about/guidelines.xhtml

migration.

Strengths and limitations of this study

- Data are from a 2014 nationally representative cross-sectional sample of internal migrants in China eight years ago, and a two-part model is used.
- Sensitivity analyses are conducted on a total sample and two subsamples to get robust results.
- The outcomes related to insurance reimbursement were self-reported, leading to measurement bias.
- Health status may influence health care utilization as well as migration status.

Keywords: migrant, health insurance, health equity, benefits, China

For peer review only - http://bmjopen.bmj.com/site/about/guidelines.xhtml

INTRODUCTION

Over the past decades, China has been experiencing rapid urbanization and internal migration[1]. Domestic (internal) migrants who lived outside their place of origin reached 375 million in 2020[2]. However, migrants faced huge barriers to obtaining health insurance where they lived[3-4]. Research has shown that migrants were less likely to be covered by social health insurance than permanent residents[4-5]. Although China established a national social health insurance system and made efforts to make the social health insurance system portable, this system was quite fragmented and administrated locally, leading to considerable variation in insurance benefits across health insurance programs and regions[6]. The unequal benefits in insurance might be amplified among the migrant population for the following reasons.

Firstly, the social health insurance system consisted of three separate insurance programs designed based on citizen's residence registration status and employment status[5]: the New Rural Cooperative Medical Scheme (NCMS) covering the registered rural residents, the Urban Employee Basic Medical Insurance (UEBMI) covering urban employees only, and the Urban Resident Basic Medical Insurance (URBMI) covering urban non-working residents[6-8]. UEBMI was jointly financed by employers and employees, while URBMI and NCMS were chiefly financed by general taxes and individual premiums. Since 2016, integrating URBMI and NCMS to Urban-Rural Resident Basic Medical Insurance (URRBMI) has piloted in some provinces and gradually expands nationally, although it is still administrated locally[9]. Subsidies from the government accounted for up to 70% of the URBMI and NCMS funds. Among the three insurance programs, UEBMI offers the most generous benefits package that provides reimbursement for both outpatient and inpatient services with high rates. URBMI and NCMS mainly reimburse for inpatient services[10]. To promote urbanization, the Chinese government issued policies to allow migrants to enroll in UEBMI or URBMI depending upon their employment status[11]. Thus, migrants may be enrolled in any of the three insurance programs, which may result in the variation in insurance benefits among migrants.

Secondly, the social health insurance programs are administrated and financed by local county or city government[12]. Each county or city designs its benefits package, which mainly covers health services delivered within the county/city and generally does not reimburse health services delivered outside the county/city[10]. Even if some

For peer review only - http://bmjopen.bmj.com/site/about/guidelines.xhtml

counties/cities cover health services provided outside the county/city, the lack of insurance portability across regions could create an additional barrier for insurance enrollees to receive benefits. Migrants may enroll in health insurance at one place but move to another and receive health services at this new place. They need to go back to where they enrolled to get insurance reimbursement. To address the insurance reimbursement issue for migrants, China promoted immediate reimbursement for cross-province medical care in 2014[13]. However, the immediate reimbursement policy was only used for inpatient services, and immediate reimbursement for outpatient services was piloted in a few regions in 2020[14]. Despite these policies, the separation between the location of health care use and health insurance coverage are still barriers to equity benefits[15].

Previous benefit analyses among the general population showed that government subsidies for social health insurance were pro-rich for inpatient and outpatient services from 2003 to 2013 in China, although the inequity in benefit distribution had been narrowed[16-18]. Evidence from URBMI (between 2007 and 2011)[19] and the social health insurance (from 2014 to 2016)[20] revealed that the lower-income groups benefited less than the higher-income groups. The poorest groups within URBMI and NCMS were consistently more likely to forego hospitalization services recommended by doctors than their wealthier counterparts between 2008 and 2018[9]. With the fragmented feature and increased benefit disparities, social health insurance reinforced the existing rural-urban inequity and generated a new inequity between urban residents and migrants who lived in urban areas[8]. In recent years, more literature documented barriers to preventing migrants from gaining access to health care[21-22] and health insurance had little influence on healthcare utilization among internal migrants[22-24]. A study by Li et al. further concluded that NCMS did not play a significant role in reducing out-of-pocket payments for elderly migrants between 2005 and 2014[25]. In addition to these disparities, there is a unique contributor to health inequity among migrants – the scope of migration – where they migrated to. However, no literature has focused on the benefit distribution of social health insurance by the scope of migration for the enormous migrant population in China.

Using the 2014 China National Internal Migrants Dynamic Monitoring Survey, we conducted a benefit analysis of social health insurance among a representative sample of migrants in China. This study was the first to assess the benefit distribution by the

For peer review only - http://bmjopen.bmj.com/site/about/guidelines.xhtml

geographic scope of migration and health insurance programs. We aimed to generate new evidence on China's continuously changing health insurance system and provide policy implications for other developing countries thriving to achieve universal health coverage under rapid urbanization.

METHODS

Data and Study Design

Data used in this analysis were from the 2014 China National Internal Migrant Dynamic Monitoring Survey. The survey was conducted by the National Health and Family Planning Commission of China in May 2014. This was a national cross-sectional survey representing 15-59-year-old internal migrants who have lived in a city of a new residence for more than one month but do not have a "Hukou" of the city (registered resident certificate).

In this survey, a stratified multi-stage random sampling method by Probability Proportional to Size (PPS) was employed, and the annual national data on internal migrants from each province in 2013 was considered as the primary sampling frame. A total of 348 cities from 32 provincial units in China were surveyed. Within each city, townships were randomly selected and followed by neighborhoods using the PPS. And then, in each neighborhood, 20 internal migrants were randomly selected to participate in the survey, finally reaching a total sample of 200,937 respondents. The face-to-face interview was conducted by trained interviewers using a structured questionnaire. Informed consent was sought from the study respondents.

Questionnaires included demographic information, family structures, socioeconomic status, migration characteristics, health insurance, health care services, and family planning services. In this study, we focused on internal migrants who used inpatient care in the city of new residence during the previous year of the survey and analyzed the benefits they received from their social health insurance. Thus, our sample comprised internal migrants who used inpatient care services in the city of a new residence and had social health insurance, with a sample size of 1165.

Measurements

In this analysis, health insurance benefits were measured by three outcomes: the probability of receiving reimbursements from social health insurance, the total

For peer review only - http://bmjopen.bmj.com/site/about/guidelines.xhtml

161 amount of reimbursement received, and the percentage of reimbursements of total health care expenditures (reimbursement ratio). In the survey, we identified the first outcome by a multiple-choice question, "where did you receive reimbursement for your last hospitalization this year." Answers included: allowance from NCMS, allowance from UEBMI, the employer, the NCMS office, the local health centers, the commercial insurance, allowance from the Family planning operation, the Family planning operation, and else. We recognized those who answered only "the commercial insurance" or "else" or both "the commercial insurance" and "else" as receiving no reimbursement from social health insurance. We identified the second outcome by the question "how much reimbursement did you receive from social health insurance." We identified the last outcome by the question for the second outcome and the question "how much did you cost in total."

Our primary predictors of interest were social health insurance programs and the geographic scope of migration. Social health insurance programs included UEBMI, URBMI, and NCMS. Since the social health insurance programs are administrated and financed by county or city, we hypothesized there are benefit disparities for migrants rooted in the separate administration of insurance programs. Therefore, our study categorized migrants according to their scope of migration, which can capture the degree of separate administration of insurance programs. The geographic scope of migration was categorized into three subgroups: migration across counties within a city, migration across cities within a province, and migration across provinces (Under China's administrative division, a county is smaller than a city).

Controlled variables included demographic characteristics, socioeconomic status, other migration characteristics, and the facility level for hospitalization. Demographic characteristics included gender, age, and marital status. Marital status was measured by a binary variable indicating whether the respondent was married or not married (e.g., widowed, divorced, or never married). Socioeconomic status was measured by educational attainment, monthly household income per capita, whether the respondent had a job, whether the respondent had rural "Hukou," and whether the respondent lived in urban areas. Educational attainment was categorized into four subgroups: primary school and below, junior high school, senior high school, and college degree and above. "Hukou" represents the record in the residency registration system in

For peer review only - http://bmjopen.bmj.com/site/about/guidelines.xhtml

China; people can be registered as having either a rural or urban “Hukou” at birth and cannot be easily changed throughout their lifetime[26]. Other migration characteristics were measured by migration reasons and duration. The reasons for migration included seeking jobs, family members following them to migrate, or other reasons. Migration duration was categorized into four groups: less than one year; one to five years; five to ten years; and ten years and above. Finally, the hospitalization facility-level included primary care facility, secondary hospital, tertiary hospital, and private hospital.

Statistical analysis

We first described the general characteristics of our study sample. Chi-square test and one-way variance analysis were used to compare the differences in the probability of receiving reimbursement and the amount and ratio of the reimbursement according to the geographic scope of migration.

Since there were many “zero observations” – patients who used inpatient care but received no reimbursement, we used the two-part model to estimate the benefits migrants received from the social health insurance, which can be expressed as follows:

$$\Pr\left[(\text{Reimburse amount})_i > 0 \;/\; (\text{Migration scope})_i\right] = \Phi[\beta * (\text{Migrantion scope})_i + \eta * X_i + \mathrm{e}_i] \qquad (1)$$

$$log[(\text{Reimburse amount})_i / \; (\text{Reimburse amount})_i > 0, (\text{Migration scope})_i, \mathrm{X}_i] = \theta * (\text{Migration scope})_i + \gamma * X_i + \psi_i \qquad (2)$$

$$(\text{Reimburse ratio})_i / \; (\text{Reimburse amount})_i > 0, (\text{Migration scope})_i, \mathrm{X}_i = \varepsilon * (\text{Migrantion scope})_i + \delta * X_i + \alpha_i \qquad (3)$$

Where$(Reimburse\ amount)_i$ and $(Reimburse\ ratio)_i$are the reimbursement amount and ratio received by individual i. $(Migration\ scope)_i$ is a set of dummies representing migration scopes of individual I, and migrants who migrated across counties within a city are taken as the reference group. The parameters β, θ, and ε, the key coefficients of interest, identify the association between migration scope and the probability of receiving reimbursement, the amount and ratio of the reimbursement conditional on reimbursement received, respectively. X_i is a vector of control variables, including social health insurance coverage, demographic characteristics,

For peer review only

For peer review only - http://bmjopen.bmj.com/site/about/guidelines.xhtml

socioeconomic status, other migration characteristics, and the facility level for hospitalization.

The above two-part model assumes that the benefits migrants received from social health insurance were determined by two separate decision-making processes: equation (1), the ‘participation equation,’ captures the fundamental difference between the respondents who received reimbursements from social health insurance and those who did not; as the ‘intensity equation,’ equation (2) or equation (3) characterizes the determinants of the amount and ratio of the reimbursement received among those who received reimbursements. In equation (2) and equation (3), the amount and ratio of the reimbursement fit the Gamma distribution, and the logarithm transformation was taken on the reimbursement amount to reduce the impact of extreme values. Following the previous study [e.g., Jan pan, Sen Tian, Qin Zhou, and Wei Han (2016)[19], we estimated equation (1) with the Probit Model, and equation (2) and equation (3) with the Generalized Linear Model (GLM), respectively. Marginal effects with standard errors were reported.

All the analyses were conducted for the total sample, rural social insurance sample (the NCMS subsample), and urban social insurance sample (the URBMI & UEBMI subsample), respectively. All analyses were performed using STATA 12.0 (StataCorp LP, College Station, TX, USA).

Patient and public involvement

No patients were actively involved in setting the research question, outcome measures nor involved in the design of the study. Patients were not involved in interpretation or write up of the results, nor are there plans for the results to be disseminated to the patient community affected by this research.

Results

Characteristics of the study sample

Table 1 presents the descriptive statistics for our study sample. Of the 1165 respondents, 66.70% enrolled in NCMS, and 23.00% and 10.30% enrolled in UEBMI and URBMI, respectively. The average expenditures per inpatient stay were 10,366 Chinese Yuan (=1,567 US dollars), and there was a small difference (about 500 Yuan ,

For peer review only - http://bmjopen.bmj.com/site/about/guidelines.xhtml

P=0.643) in inpatient expenditure between the NCMS and URBMI & UEBMI subsample. 66.78% of respondents who used inpatient care received reimbursement from social health insurance, with 60.49% for NCMS enrollees and 79.38% for URBMI & UEBMI enrollees. Among the respondents who received reimbursement, the average amount and ratio of the reimbursement received were 5,506 Yuan (=832 US dollars) and 46.77%. The average amount and ratio of the reimbursement received for NCMS enrollees were much smaller than those for URBMI & UEBMI enrollees.

The average age and monthly household Income per capita of the respondents were 38 years old and 2,256 Yuan. Less than half of the respondents were female. Most of them were married (89.27%), had an education level of high school or below (89.70%), had rural "Hukou" (87.81%), owned a job (79.57%), and lived in urban areas (69.36%). Nearly half of the respondents migrated across provinces, while those who migrated across cities but within a province and those who migrated across counties but within a city were 28.76% and 25.41%, respectively. 84.21% of respondents migrated for better job opportunities, and 87.38% of respondents had lived in the city of a new residence for more than one year. Most respondents (80.51%) chose inpatient care at secondary and tertiary hospitals instead of primary care facilities.

Table 1 Characteristic of the study sample, n (%)

Variables	Total sample (N=1165)	NCMS subsample (N=777)	URBMI&UEBMI subsample (N=388)
Total expenditure per inpatient stay (Yuan) *	10366.05 (±17549.73)	10197.45 (±18374.85)	10704.99 (±15778.28)
Probability of receiving reimbursement	778(66.78)	470(60.49)	308(79.38)
Reimbursement amount received (Yuan) *	5506.16 (±9761.79)	5128.44 (±10838.10)	6049.14 (±7952.00)
Reimbursement ratio received (%) *	46.77(±19.91)	39.41(±17.86)	57.35(±17.87)
Social health insurance programs			
NCMS	777(66.70)	-	-
URBMI	120(10.30)	-	120(30.93)
UEBMI	268(23.00)	-	268(69.07)
Age (Years) *	37.65(±9.75)	37.79(±9.80)	37.37(±9.65)
Female	532(45.67)	361(46.46)	171(44.07)
Married	1040(89.27)	705(90.73)	335(86.34)
Education attainment			
Primary school and below	279(23.95)	218(28.06)	61(15.72)
Junior high school	554(47.55)	416(53.54)	138(35.57)
Senior high school	212(18.20)	118(15.19)	94(24.23)
College and above	120(10.30)	25(3.22)	95(24.48)
Monthly household income per capita	2256.43(±2092.85)	1999.55(±1687.47)	2770.86(±2658.26)

For peer review only - http://bmjopen.bmj.com/site/about/guidelines.xhtml

(Yuan) *			
Having any job	927(79.57)	610(78.51)	317(81.70)
Rural Hukou	1023(87.81)	777(100.00)	267(68.81)
Living in an urban area	808(69.36)	502(64.61)	306(78.87)
Geographic scope of migration			
Across counties within a city	296(25.41)	216(27.80)	80(20.62)
Across cities within a province	335(28.76)	214(27.54)	121(31.19)
Across provinces	534(45.84)	347(44.66)	187(48.20)
Reasons for migration			
Seeking jobs	981(84.21)	642(82.63)	339(87.37)
Family members following migrants	146(12.53)	115(14.80)	31(7.99)
Other reasons	38(3.26)	20(2.57)	18(4.64)
Migration duration (Years)			
0~1	147(12.62)	117(15.06)	30(7.73)
1~5	484(41.55)	305(39.25)	179(46.13)
5~10	272(23.35)	181(23.29)	91(23.45)
10 +	262(22.49)	174(22.39)	88(22.68)
Facility level of hospitalization			
Primary care facility	131(11.24)	90(11.58)	41(10.57)
Secondary hospital	400(34.33)	282(36.29)	118(30.41)
Tertiary hospital	538(46.18)	340(43.76)	198(51.03)
Private hospital	96(8.24)	65(8.37)	31(7.99)

Note: * mean (± Standard Deviation). NCMS: New Rural Cooperative Medical Scheme; URBMI: Urban Resident Basic Medical Insurance; UEBMI: Urban Employee Basic Medical Insurance.

We compared the characteristics of the study sample by the scope of migration (Appendix Table 1). The demographic and socioeconomic characteristics of migrants were similar across three scopes of migration, including age, sex, marriage status, education, job status, Hukou status, reasons for migration, migration duration, and health insurance programs they enrolled in. The Only exception was income and living areas. Migrants across provinces had more income and were less likely to live in urban areas than other groups.

Table 2 summarizes the total expenditures per inpatient stay and the probability of benefiting from social health insurance. It also presents the reimbursement amount and ratio received among the benefit recipients according to the geographic scope of migration. The univariate analysis showed that the broader the migration scope, the

For peer review only

For peer review only - http://bmjopen.bmj.com/site/about/guidelines.xhtml

lower the probability that migrants would receive reimbursements; but among those who received reimbursements, those who migrated across cities or across provinces received larger amounts of reimbursement than those who migrated within a city. There was no significant difference in total expenditure and reimbursement ratio by the geographic scope of migration.

Table 2 Benefit distribution of social health insurance by geographic scope of migration

Variables	Across counties within a city	Across cities within a province	Across provinces	p-value
Total expenditure per inpatient stay (Yuan)	10326.81 (14079.35)	14458.96 (27139.10)	12771.68 (20428.78)	0.354
Probability of receiving reimbursement (%)	81.76 (38.69)	69.85 (45.96)	56.55 (49.62)	<0.001
Reimbursement amount received (Yuan)	4402.12 (6541.01)	6706.32 (13681.56)	5495.89 (8105.80)	0.056
Reimbursement ratio received (%)	44.75 (18.17)	47.76 (20.51)	47.72 (20.79)	0.195

Note: Standard Deviations are reported in parentheses.

Association between insurance programs, migration scope, and social health insurance benefits

Table 3 reports the association between insurance programs, migration scope, other factors, and social health insurance benefits, estimated from a two-part model. Compared with NCMS enrollees, URBMI or UEBMI enrollees were more likely to receive reimbursement, and among the benefit recipients, urban insurance enrollees received a larger reimbursement amount and ratio. The probability of receiving reimbursement for UEBMI enrollees was 37.5% (P<0.01) higher than that for the NCMS enrollees. Among insurance benefit recipients, UEBMI enrollees received 42.8% (P<0.01) more reimbursement amount and 20.1% (P<0.01) higher reimbursement ratio than NCMS enrollees.

According to the association between insurance benefits and migration scope, the geographic scope of migration reduced the probability of receiving reimbursement and the reimbursement ratio but increased the reimbursement amounts they received. Specifically, the probability of receiving reimbursement for those who migrated across cities and provinces was 14.7% and 26.0%, respectively, significantly lower than those who migrated within a city (P<0.01). However, they received 33.4% and

For peer review only - http://bmjopen.bmj.com/site/about/guidelines.xhtml

27.2% higher reimbursement amounts than those who migrated within a city (P<0.01). And those who migrated across provinces had the lowest reimbursement ratio although not reaching significance level.

In addition, there was no significant difference in insurance benefits by age, gender, marriage status, education, Hukou status, and migration duration. Income had no significant influence on the probability and ratio of reimbursement but significantly increased the reimbursement amount. Having jobs significantly decreased the reimbursement amount, whereas living in urban areas significantly increased the probability of receiving reimbursement by 6.3% compared with living in suburban areas. Compared with migration for seeking jobs, family members following migrants significantly increased the reimbursement amount. The higher the level of health care facility, the greater the reimbursement amount, but the lower the reimbursement ratio.

Table 3 Association between insurance programs, migration scope, other factors, and social health insurance benefits

Variables	Probit Model		Generalized Linear Model			
	Probability of getting reimbursements		Reimbursement amount		Reimbursement ratio	
	Coefficient	S.E.	Coefficient	S.E.	Coefficient	S.E.
Social health insurance (referred to NCMS)						
URBMI	0.021	0.049	0.222	0.136	0.147**	0.031
UEBMI	0.375**	0.044	0.428**	0.094	0.201**	0.023
Age (years)	0.010	0.012	0.026	0.030	0.006	0.006
Age^2 (years)	0.000	0.000	0.000	0.000	0.000	0.000
Female	0.024	0.031	-0.004	0.078	0.018	0.016
Married	0.063	0.051	-0.037	0.146	0.010	0.032
Education attainment (referred to primary school and below)						
Junior high school	0.039	0.038	0.108	0.099	0.014	0.020
Senior high school	0.081	0.050	0.014	0.125	0.027	0.026
College and above	-0.038	0.068	-0.015	0.173	0.032	0.039
Monthly household income per capita (referred to first quintile)						
2nd quintile	0.062	0.048	0.328**	0.124	-0.029	0.026
3rd quintile	-0.033	0.051	0.014	0.139	-0.041	0.028
4th quintile	0.047	0.049	0.179	0.124	-0.031	0.026
5th quintile	0.029	0.053	0.336*	0.137	-0.023	0.030
Having any job	-0.070	0.042	-0.247*	0.109	0.010	0.022
Rural hukou	0.088	0.053	-0.106	0.126	0.000	0.031
Living in an urban area	0.063*	0.032	-0.012	0.088	0.004	0.017

For peer review only

For peer review only - http://bmjopen.bmj.com/site/about/guidelines.xhtml

Geographic scope of migration (referred to migration across counties within a city)						
Across cities within a province	-0.147**	0.041	0.334**	0.093	-0.019	0.019
Across provinces	-0.260**	0.038	0.272**	0.093	-0.032	0.019
Reasons for migration (referred to Seeking jobs)						
Family members following migrants	-0.007	0.050	0.553**	0.137	0.047	0.027
other reasons	-0.025	0.087	-0.224	0.207	0.024	0.048
Migration duration (referred to 0~1)						
1~5	-0.014	0.045	0.078	0.125	-0.032	0.026
5~10	0.017	0.051	0.162	0.135	-0.022	0.028
10 +	-0.011	0.052	0.085	0.140	-0.040	0.029
Facility level of hospitalization (referred to primary care facility)						
Secondary hospital	-0.031	0.048	0.603**	0.139	-0.046	0.032
Tertiary hospital	0.083	0.048	1.065**	0.135	-0.083**	0.031
Private hospital	-0.156*	0.063	0.092	0.188	-0.027	0.043
Observations	1165		663		663	

Note: ** and * denote statistical significance at 1and 5% level, respectively.

S.E.: Standard Error. NCMS: New Rural Cooperative Medical Scheme; URBMI: Urban Resident Basic Medical Insurance; UEBMI: Urban Employee Basic Medical Insurance.

Considering the differences in reimbursement policy between NCMS and urban health insurance, we further conducted the above regressions among the subsamples of NCMS enrollees and URBMI & UEMBI enrollees (Table 4). The relationships between migration scope and the reimbursement probability and amount did not change, but they differed with the reimbursement ratio. Those who migrated more broadly had a significantly lower reimbursement ratio among NCMS enrollees, while that among URBMI & UEMBI enrollees was obviously higher.

Table 4 Association between migration scope and benefit of social health insurance programs

Variables	Probit Model		Generalized Linear Model			
	Probability of getting reimbursements		Reimbursement amount		Reimbursement ratio	
	Coefficient	S.E.	Coefficient	S.E.	Coefficient	S.E.
**NCMS subsample**						
Geographic scope of migration (referred to migration across counties within a city)						
Across cities within a province	-0.139**	0.052	0.410**	0.120	-0.044	0.023
Across provinces	-0.333**	0.048	0.282*	0.118	-0.055*	0.024
**URBMI & UEBMI subsample**						

For peer review only - http://bmjopen.bmj.com/site/about/guidelines.xhtml
For peer review only

Geographic scope of migration (referred to migration across counties within a city)						
Across cities within a province	-0.135*	0.061	0.339*	0.154	0.073*	0.033
Across provinces	-0.118	0.061	0.285	0.152	0.039	0.033

Note: All models included confounding factors in Table 3. ** and * denote statistical significance at 1% and 5% levels.

S.E.: Standard Error. NCMS: New Rural Cooperative Medical Scheme; URBMI: Urban Resident Basic Medical Insurance; UEBMI: Urban Employee Basic Medical Insurance.

Reasons for not receiving reimbursement from social health insurance

We further investigated why migrants did not receive reimbursement from their social health insurance. Figure 1 showed that the need or plan to go back to hometown to get reimbursement was the main reason for not getting reimbursement, accounting for 66.5%, followed by a lack of knowledge about the reimbursement process (16.7%) and the policy coverage issues (10.6%). Figure 2 compares the proportion of not receiving reimbursement due to the need or plan to go back to their hometown by migration scope. The broader the migration scope, the higher the likelihood that migrants did not receive reimbursement because they must get it later from their hometowns.

Discussion

We found that the probability of receiving reimbursement and the reimbursement ratio for migrants were far lower than those for the general population. Only 60% of migrants who were NCMS enrollees received reimbursement from NCMS, which was 30% lower than that of general NCMS enrollees (91.1% in 2013), and the reimbursement ratio was 10% lower than that of general NCMS enrollees (39.4% vs. 50.1% in 2013)[27]. For URBMI & UEBMI enrollees, the probability of receiving reimbursement among migrants was about 10% lower than that of the general population (79.4% vs. 95.3% for UEBMI and 88.7% for URBMI), although there is a slight difference in the reimbursement ratio between the two groups (around 60%)[27]. A previous study also pointed out that migrants only partially benefited from health insurance coverage[24]

For peer review only

For peer review only - http://bmjopen.bmj.com/site/about/guidelines.xhtml

The results should be understood in the China-specific context. In China, migrants face more challenges in obtaining insurance reimbursement than non-migrants. There have been two common approaches to reimburse health care services (immediate reimbursement and later reimbursement)[28]. Immediate reimbursement means that the insured patient gets reimbursement immediately for the treatment and only pays out-of-pocket for the copay or coinsurance rate, whereas later reimbursement means that the insured patient pays the total expenditures out-of-pocket up-front and gets reimbursement later from their health insurance[28]. Local residents usually get reimbursement immediately, but migrants generally receive reimbursement later as they must travel back to their hometown where they enroll in social health insurance. Our study showed that up to 22% of migrants reported that they did not get reimbursement because they needed to go back to their hometowns. In addition, research has shown that many services were not reimbursed, and the reimbursement process for migrants was much more complex than that for local residents[29]. One study showed that compared with local residents, more migrants were treated in a hospital outside the NCMS designated network, and thus their healthcare use was less likely to be covered by NCMS[29].

Our main findings showed inequity in insurance benefits among migrants by migration scope. Although the scope of migration was associated with a larger reimbursement amount per inpatient stay, it was significantly associated with a lower probability of receiving reimbursement. All three social health insurance programs in China were administered, financed, and operated by local county or city governments. Each county or city designed its benefits packages and made the benefit localized[10][30], limiting individual coverage choices outside the local region. This poses a challenge for internal migrants who typically use health care in the city of new residence but may enroll in health insurance at their hometown according to their residence ("Hukou") status. The separation between where health care is received and where health insurance is administrated provided an additional hurdle for the internal migrants to receive benefits from their social health insurance. It became even more difficult when they lived far away from their hometowns. The localized administration of social health insurance and the later reimbursement approach contributed jointly to the inequity benefit for migrants[31-32]. To resolve this inequity, China has strived to improve the portability of social health insurance through policy

For peer review only - http://bmjopen.bmj.com/site/about/guidelines.xhtml

development and the construction of a national health insurance information platform. However, till now, the information platform among different provinces has not been fully interconnected[33]. Moreover, these efforts mainly focus on insurance reimbursement for cross-province inpatient care rather than outpatient care.

Another important finding of this study was that migrants who got reimbursement received larger reimbursement amounts if they migrated more broadly. Compared to migration within a city, migration across cities or provinces were significantly associated with about 30% higher reimbursement amount per inpatient stay. This is mainly attributed to higher health care costs in larger and more affluent regions where migrants preferred to locate. However, these reimbursements were paid primarily by health insurance funds in smaller counties/cities which were less affluent. This may pose financial difficulties for local governments in those out-migration regions if the current barriers to reimbursement are eliminated, and there will be more significant amounts of insurance funds flowing into the health care system in more prosperous in-migration regions, which will worsen the already skewed regional inequity in economic development and health. Therefore, the inverse subsidies of health insurance funds from the less-developed out-migration regions to the highly-developed in-migration regions have become an ongoing challenge and policy dilemma faced by countries like China that has experienced mass domestic migration. Strategies that control health expenditures inflation and allow the central government to redistribute welfare fund to less-developed regions may be warranted to address the financial difficulties faced by out-migration regions[6].

In addition to the above inequity related to migration, there were two other types of inequity in benefits due to the fragmented social insurance system and income inequity. Although China has almost achieved universal insurance coverage through social health insurance expansion[6], migrants who enrolled in UEBMI and URBMI benefited more than those enrolled in NCMS[10][34]. Several national studies found that among the general population, UEBMI enrollees had a higher benefit level than those covered by URBMI or NCMS[35-36]. Even for the same condition – tuberculosis inpatient care- the reimbursement rate was the highest for UEBMI enrollees, followed by URBMI and NCMS enrollees in 2012[34]. Income inequity also explains some benefit inequities. Among the benefit recipients, we found that migrants with higher income received greater reimbursement amounts, which was consistent with previous

For peer review only - http://bmjopen.bmj.com/site/about/guidelines.xhtml

studies among the general population[16][19][37]. On average, the higher-income group tended to have higher inpatient expenditure, and it was not surprising that they also received greater reimbursement amount[38].

This study contained some limitations. First, the outcomes related to reimbursement were self-reported, leading to measurement bias. Future research will use health insurance claims data to minimize this bias. Second, health status may influence health care utilization and whether a person chooses to migrate. Studies have detected the healthy migrant effect for internal migrants in China, showing that healthier people were more likely to migrate and move farther away from home[39]. Unfortunately, there was no measurement of the health status of migrants in this dataset. Third, we could not accurately distinguish the administrative site of social health insurance, which may affect the insurance benefit for internal migrants. To reduce this bias, we conducted the analysis separately on two subsamples of NCMS enrollees and UEBMI & URBMI enrollees. Lastly, the 2014 China National Internal Migrant Dynamic Monitoring Survey is a dataset eight years ago, which may limit its implications for current issues.

Conclusions

This study has important policy implications not only for China but also for other developing countries that experience rapid urbanization and internal migration. In some developing countries (e.g., Mexico), health insurance programs are administrated locally and separated across regions[40-41], hindering the insurance benefits when people migrate across regions. When their internal migrants move beyond the administrated region, they will face the same problem as the Chinese migrants.

In China, the broader the migration scope, the lower the probability of receiving reimbursements from the social insurance and the reimbursement ratio; but among those who received reimbursements, the broader the migration scope, the larger amounts they were reimbursed for health care use. This unequal benefit distribution may be attributed to the fragmented insurance design which relies on localized administration and patients paying services up-front and requesting reimbursement

For peer review only - http://bmjopen.bmj.com/site/about/guidelines.xhtml

later. To improve the equity in social insurance benefits, China has been promoting the portability of social health insurance, immediate reimbursement for inpatient care utilized across regions, and a more integrated health insurance system. Efforts should also be made to control inflation of health care expenditures and prevent inverse government subsidies from out-migration regions to in-migration regions.

For peer review only

For peer review only - http://bmjopen.bmj.com/site/about/guidelines.xhtml

Statements

Contributorship

ZH designed the study and drafted the manuscript. HW conducted the literature review, analyzed the data, and co-drafted the manuscript. DL and DZ revised the manuscript. All authors read and approved the final manuscript being submitted.

Funding Statement

This work was supported by the National Natural Science Foundation of China (No. 71874034) and the National Key R&D Program of China (No. 2018YFC1312600 and 2018YFC1312604). The funders had no role in study design, data collection and analysis, preparation of the paper, or the decision to publish.

Competing of Interests

None declared.

Ethics approval

The data used in this paper were publicly available, provided by the National Health and Family Planning Commission of China. Thus, there is no necessary to conduct an additional ethics approval.

Data sharing

The data used in this paper were provided by the National Health and Family Planning Commission of China. We signed a legally binding agreement with the Commission that we would not share the original data with any third parties. Although researchers who are interested can apply to get access to the data at: http://www.chinaldrk.org.cn and Email: ldrkzxsj@163.com, it is up to the National Health and Family Planning Commission to make the final decision.

For peer review only - http://bmjopen.bmj.com/site/about/guidelines.xhtml

References

1 P Gong, S Liang, EJ Carlton, et al. Urbanisation and health in China. *The Lancet* 2012;9818(379):843-52.

2 National Bureau of Statistics. Bulletin of the Seventh National Population Census (No. 7). In, 2021.

3 JX Fan, M Wen, L Jin, G Wang. Disparities in Healthcare Utilization in China: Do Gender and Migration Status Matter? *J Fam Econ Iss* 2013;34(1):52-63.

4 S Wang, A Liu, W Guo. Public and Commercial Medical Insurance Enrollment Rates of Rural-to-Urban Migrants in China. *Frontiers in Public Health* 2021;9.

5 Y Jin, Z Hou, D Zhang. Determinants of Health Insurance Coverage among People Aged 45 and over in China: Who Buys Public, Private and Multiple Insurance. *Plos One* 2016;11(8):e161774.

6 Q Meng, H Fang, X Liu, B Yuan, J Xu. Consolidating the social health insurance schemes in China: towards an equitable and efficient health system. *Lancet* 2015;386(10002):1484-92.

7 Z Hou, E Van de Poel, E Van Doorslaer, B Yu, Q Meng. EFFECTS OF NCMS ON ACCESS TO CARE AND FINANCIAL PROTECTION IN CHINA. *Health Econ* 2014;23(8):917-34.

8 X Huang. Expansion of Chinese Social Health Insurance: who gets what, when and how? *J Contemp China* 2014;23(89):923-51.

9 X Yan, Y Liu, M Cai, et al. Trends in disparities in healthcare utilisation between and within health insurances in China between 2008 and 2018: a repeated cross-sectional study. *Int J Equity Health* 2022;21(1).

10 Q Wang, D Zhang, Z Hou. Insurance coverage and socioeconomic differences in patient choice between private and public health care providers in China. *Soc Sci Med* 2016;170:124-32.

11 Ministry of Human Resources and Social Security of the People's Republic of China. Interim Measures for the Transferability of Basic Medical Insurance Account among Internal Migrants. In, 2009.

12 M Ramesh, X Wu, AJ He. Health governance and healthcare reforms in China. *Health Policy Plann* 2014;29(6):663-72.

13 General Office of the State Council. Notice on key missions for deepeng health system reform in 2014. In, 2014.

14 National Healthcare Security Administration. Notice on Promoting the Pilot Program of Cross-Provincial Direct Settlement of Outpatient Expenses. In, 2020.

15 S Chen, Y Chen, Z Feng, et al. Barriers of effective health insurance coverage for rural-to-urban migrant workers in China: a systematic review and policy gap analysis. *Bmc Public Health* 2020;20(1).

16 M Chen, G Fang, L Wang, et al. Who Benefits from Government Healthcare Subsidies? An Assessment of the Equity of Healthcare Benefits Distribution in China. *Plos One* 2015;10(3):e119840.

17 M Chen, AJ Palmer, L Si. Assessing equity in benefit distribution of government health subsidy in 2012 across East China: benefit incidence analysis. *Int J Equity Health* 2016;15(1).

18 L Si, M Chen, AJ Palmer. Has equity in government subsidy on healthcare improved in China? Evidence from the China’s National Health Services Survey. *Int J Equity Health* 2017;16(1).

19 J Pan, S Tian, Q Zhou, W Han. Benefit distribution of social health insurance: evidence from china’s urban resident basic medical insurance. *Health Policy Plann* 2016;31(7):853-59.

20 L Diao, Y Liu. Inequity under equality: research on the benefits equity of Chinese basic medical

For peer review only - http://bmjopen.bmj.com/site/about/guidelines.xhtml

insurance. *Bmc Health Serv Res* 2020;20(1).
21 X Ke, L Zhang, Z Li, W Tang. Inequality in health service utilization among migrant and local children: a cross-sectional survey of children aged 0–14 years in Shenzhen, China. *Bmc Public Health* 2020;20(1).
22 F Zhang, X Shi, Y Zhou. The Impact of Health Insurance on Healthcare Utilization by Migrant Workers in China. *Int J Env Res Pub He* 2020;17(6):1852.
23 H Wang, D Zhang, Z Hou, F Yan, Z Hou. Association between social health insurance and choice of hospitals among internal migrants in China: a national cross-sectional study. *Bmj Open* 2018;8(2):e18440.
24 X Qin, J Pan, GG Liu. Does participating in health insurance benefit the migrant workers in China? An empirical investigation. *China Econ Rev* 2014;30:263-78.
25 J Li, Y Huang, S Nicholas, J Wang. China's New Cooperative Medical Scheme's Impact on the Medical Expenses of Elderly Rural Migrants. *Int J Env Res Pub He* 2019;16(24):4953.
26 Z Zhang, DJ Treiman. Social origins, hukou conversion, and the wellbeing of urban residents in contemporary China. *Soc Sci Res* 2013;42(1):71-89.
27 National Health Commission of the People's Republic of China. An Analysis Report of National Health Services Survey in China，2013. Beijing: Peking Union Medical College Press 2015.
28 H Zhong. Effect of patient reimbursement method on health-care utilization: evidence from China. *Health Econ* 2011;20(11):1312-29.
29 P Qiu, Y Yang, J Zhang, X Ma. Rural-to-urban migration and its implication for new cooperative medical scheme coverage and utilization in China. *Bmc Public Health* 2011;11:520.
30 Y Zhao, B Kang, Y Liu, et al. Health insurance coverage and its impact on medical cost: observations from the floating population in China. *Plos One* 2014;9(11):e111555.
31 Q Gao, S Yang, S Li. Labor contracts and social insurance participation among migrant workers in China. *China Econ Rev* 2012;23(4):1195-205.
32 KKF Lam, JM Johnston. Health insurance and healthcare utilisation for Shenzhen residents: a tale of registrants and migrants? 2012;12(1):868.
33 Y Chen. Study On The Optimization of Long-Distance Medical Insurance of Public Hospitals In Shanghai: Based on Holistic Governance Theory. *Shanghai Jiao Tong University*, 2020.(in Chinese)
34 Y Pan, S Chen, M Chen, et al. Disparity in reimbursement for tuberculosis care among different health insurance schemes: evidence from three counties in central China. *Infect Dis Poverty* 2016;5:7.
35 L X,J WY. Empirical Study on the Benefit Equity of Essential Health Insurance System in China. *medicine and Society* 2010;23(11):45-47.(in Chinese)
36 W Dong, AB Zwi, R Bai, C Shen, J Gao. Benefits Associated with China's Social Health Insurance Schemes: Trend Analysis and Associated Factors Since Health Reform. *Int J Env Res Pub He* 2021;18(11):5672.
37 S Yuan, C Rehnberg, X Sun, X Liu, Q Meng. Income related inequalities in New Cooperative Medical Scheme: a five-year empirical study of Junan County in China. 2014;13(1):38.
38 Z Zhou, J Gao, A Fox, et al. Measuring the equity of inpatient utilization in Chinese rural areas. *Bmc Health Serv Res* 2011;11:201.
39 Y Lu, L Qin. Healthy migrant and salmon bias hypotheses: a study of health and internal migration in China. *Soc Sci Med* 2014;102:41-48.

For peer review only - http://bmjopen.bmj.com/site/about/guidelines.xhtml

40 XY Li, Y Zhu, ZL Wu. Comparison of health financing mechanisms under the perspective of universal health coverage in developing countries. *Jiangsu J Prev Med* 2017;28(03):254-58.(in Chinese)

41 FM Knaul, E González-Pier, O Gómez-Dantés, et al. The quest for universal health coverage: achieving social protection for all in Mexico. *Lancet* 2012;380(9849):1259-79.

For peer review only

For peer review only - http://bmjopen.bmj.com/site/about/guidelines.xhtml

Figure 1. Proportions of reasons not getting reimbursement from social health insurance (%).

Figure 2. Proportion of inpatients not getting reimbursement due to the need or plan to go back to hometown for reimbursement, by geographic scope of migration (%). Proportions and 95% confidence interval are shown.

For peer review only

For peer review only - http://bmjopen.bmj.com/site/about/guidelines.xhtml

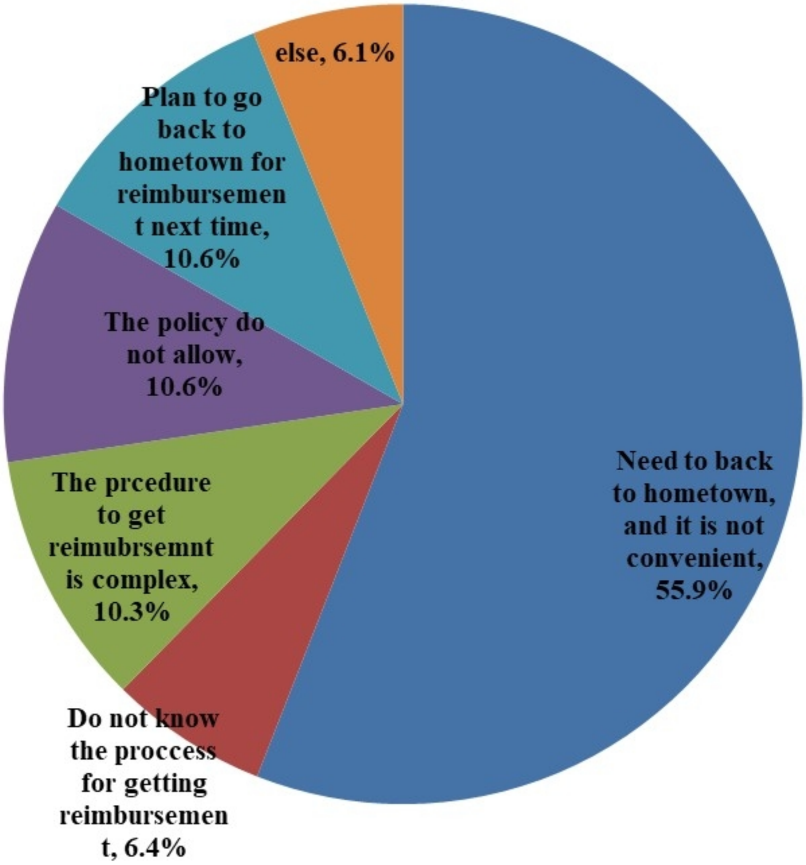

Figure 1. Proportions of reasons not getting reimbursement from social health insurance (%).

125x123mm (150 x 150 DPI)

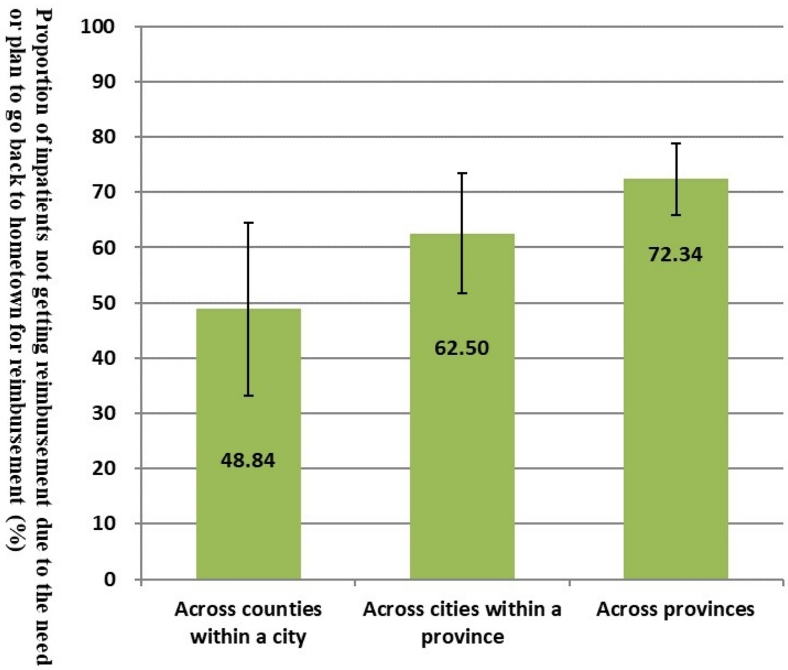

Figure 2. Proportion of inpatients not getting reimbursement due to the need or plan to go back to hometown for reimbursement, by geographic scope of migration (%). Proportions and 95% confidence interval are shown.

153x115mm (150 x 150 DPI)

Appendix Table 1 Characteristics of the study sample by migration scope

	Across counties within a city	Across cities within a province	Across provinces	F/χ2	P value
Age (Years) *	38.41(±10.35)	37.99(±9.62)	37.01 (±9.47)	2.248	0.106
Female	137(46.28%)	154(46.0%)	241(45.13%)	0.12	0.942
Married	262(88.51%)	298(88.96%)	480(89.89%)	0.424	0.809
Education attainment				6.979	0.323
Primary school and below	72(24.32%)	78(23.28%)	129(24.16%)		
Junior high school	137(46.28%)	154(45.97%)	263(49.25%)		
Senior high school	64(21.62%)	62(18.51%)	86(16.10%)		
College and above	23(7.77%)	41(12.24%)	56(10.49%)		
Monthly household income per capita (Yuan)*	1830.88(±1272.07)	1968.94(±1589.62)	2672.67(±2600.04)	20.506	0.000
Having any job	226(76.35%)	264(78.81%)	437(81.84%)	3.692	0.158
Rural Hukou	268(90.54%)	293(87.46%)	462(86.52%)	2.934	0.231
Living in an urban area	237(80.07%)	241(71.94%)	330(61.80%)	31.386	0.000
Reasons for migration				8.13	0.087
Seeking jobs	242(81.76%)	273(81.49%)	466(87.27%)		
Family members following migrants	44(14.86%)	51(15.22%)	51(9.55%)		
Other reasons	10(3.38%)	11(3.28%)	17(3.18%)		
Migration duration (Years)				10.475	0.106
0~1	43(14.53%)	32(9.55%)	72(13.48%)		
1~5	135(45.61%)	135(40.30%)	214(40.07)		
5~10	65(21.96%)	80(23.88%)	127(23.78%)		
10 +	53(17.91%)	88(26.27%)	121(22.66%)		
Social health insurance programs				8.376	0.079
NCMS	216(72.97%)	214(63.88%)	347(64.98%)		
URBMI	28(9.50%)	39(11.64%)	53(9.93%)		
UEBMI	52(17.57%)	82(24.48%)	134(25.09%)		

Note: * mean (± Standard Deviation).

For peer review only

STROBE Statement—checklist of items that should be included in reports of observational studies

	Item No.	Recommendation	Page No.	Relevant text from manuscript
**Title and abstract**	1	(*a*) Indicate the study's design with a commonly used term in the title or the abstract	Page 1, Line 2<br>Page 2, Line24	a National Cross-Sectional Study
		(*b*) Provide in the abstract an informative and balanced summary of what was done and what was found	Page 2, Line 22, Line 28-30<br>Page 2, Line 31-41	Objectives: To evaluate the benefit distribution of social health insurance among internal migrants in China.<br>Primary and secondary outcome measures: The probability of receiving reimbursements from social health insurance, the amounts and ratio of reimbursement received.<br>Results: Among migrants who utilized inpatient care in 2013……And those who migrated across provinces had the lowest reimbursement ratio although not reaching significance level.
**Introduction**				
Background/rationale	2	Explain the scientific background and rationale for the investigation being reported	Page 4, Line 64-106	Over the past decades, China has been experiencing rapid urbanization and internal migration……Despite these policies, the separation between the location of health care use and health insurance coverage

For peer review only - http://bmjopen.bmj.com/site/about/guidelines.xhtml

				are still barriers to equity benefits
Objectives	3	State specific objectives, including any prespecified hypotheses	Page 6, Line 126-132	Using the 2014 China National Internal Migrants Dynamic Monitoring Survey……We aimed to generate new evidence on China's continuously changing health insurance system and provide policy implications for other developing countries thriving to achieve universal health coverage under rapid urbanization
**Methods**				
Study design	4	Present key elements of study design early in the paper	Page 6, Line 136-141	Data used in this analysis were from the 2014 China National Internal Migrant Dynamic Monitoring Survey. The survey was conducted by the National Health and Family Planning Commission of China in May 2014. This was a national cross-sectional survey representing 15-59-year-old internal migrants who have lived in a city of a new residence for more than one month but do not have a "Hukou" of the city (registered resident certificate).

For peer review only - http://bmjopen.bmj.com/site/about/guidelines.xhtml
For peer review only

Setting	5	Describe the setting, locations, and relevant dates, including periods of recruitment, exposure, follow-up, and data collection	Page 6, Line 138-153	This was a national cross-sectional survey representing 15-59-year-old internal migrants who have lived in a city of a new residence for more than one month but do not have a "Hukou" of the city (registered resident certificate)……Questionnaires included demographic information, family structures, socioeconomic status, migration characteristics, health insurance, health care services, and family planning services.
Participants	6	(*a*) *Cohort study*—Give the eligibility criteria, and the sources and methods of selection of participants. Describe methods of follow-up<br>*Case-control study*—Give the eligibility criteria, and the sources and methods of case ascertainment and control selection. Give the rationale for the choice of cases and controls<br>*Cross-sectional study*—Give the eligibility criteria, and the sources and methods of selection of participants	Cross-sectional study<br>Page 6, Line 153-157	In this study, we focused on internal migrants who used inpatient care in the city of new residence during the previous year of the survey and analyzed the benefits they received from their social health insurance. Thus, our sample comprised internal migrants who used inpatient care services in the city of a new residence and had social health insurance, with a sample size of 1165.
		(*b*) *Cohort study*—For matched studies, give matching criteria and number of exposed and unexposed<br>*Case-control study*—For matched studies, give matching criteria and the number of controls per case	Not Applicable	

For peer review only - http://bmjopen.bmj.com/site/about/guidelines.xhtml

Variables	7	Clearly define all outcomes, exposures, predictors, potential confounders, and effect modifiers. Give diagnostic criteria, if applicable	Page 6-7, Line 159-184	In this analysis, health insurance benefits were measured by three outcomes……Controlled variables included demographic characteristics, socioeconomic status, other migration characteristics, and the facility level for hospitalization.
Data sources/ measurement	8*	For each variable of interest, give sources of data and details of methods of assessment (measurement). Describe comparability of assessment methods if there is more than one group	Page 7-8, Line 159-200	In this analysis, health insurance benefits were measured by three outcomes……Finally, the hospitalization facility-level included primary care facility, secondary hospital, tertiary hospital, and private hospital.
Bias	9	Describe any efforts to address potential sources of bias	Page 9, Line 238-240	All the analyses were conducted for the total sample, rural social insurance sample (the NCMS subsample), and urban social insurance sample (the URBMI & UEBMI subsample), respectively
Study size	10	Explain how the study size was arrived at	Page 6, Line 155-157	Thus, our sample comprised internal migrants who used inpatient care services in the city of a new residence and had social health insurance, with a sample size of 1165.

Continued on next page

For peer review only - http://bmjopen.bmj.com/site/about/guidelines.xhtml
For peer review only

Quantitative variables	11	Explain how quantitative variables were handled in the analyses. If applicable, describe which groupings were chosen and why	Page 8, Line 196-198	Migration duration was categorized into four groups: less than one year; one to five years; five to ten years; and ten years and above.
Statistical methods	12	(*a*) Describe all statistical methods, including those used to control for confounding	Page 8, Line 202-205<br><br>Page 9, Line 225-237	We first described the general characteristics of our study sample. Chi-square test and one-way variance analysis were used to compare the differences in the probability of receiving reimbursement and the amount and ratio of the reimbursement according to the geographic scope of migration.<br>The above two-part model assumes that the benefits migrants received from social health insurance were determined by two separate decision-making processes…we estimated equation (1) with the Probit Model, and equation (2) and equation (3) with the Generalized Linear Model (GLM), respectively.
		(*b*) Describe any methods used to examine subgroups and interactions	Not Applicable	
		(*c*) Explain how missing data were addressed	Not Applicable	
		(*d*) *Cohort study*—If applicable, explain how loss to follow-up was addressed<br>*Case-control study*—If applicable, explain how matching of cases and controls was addressed<br>*Cross-sectional study*—If applicable, describe analytical methods taking account of sampling strategy	Not Applicable	

For peer review only

		(*e*) Describe any sensitivity analyses	Page 9, Line 238-240	All the analyses were conducted for the total sample, rural social insurance sample (the NCMS subsample), and urban social insurance sample (the URBMI & UEBMI subsample), respectively.
**Results**				
Participants	13*	(a) Report numbers of individuals at each stage of study—eg numbers potentially eligible, examined for eligibility, confirmed eligible, included in the study, completing follow-up, and analysed	Page 9, Line 250-251	Of the 1165 respondents…
		(b) Give reasons for non-participation at each stage	Not Applicable	
		(c) Consider use of a flow diagram	Not Applicable	
Descriptive data	14*	(a) Give characteristics of study participants (eg demographic, clinical, social) and information on exposures and potential confounders	Page 9-10, Line 250-271	Table 1 presents the descriptive statistics for our study sample. Of the 1165 respondents……Most respondents (80.51%) chose inpatient care at secondary and tertiary hospitals instead of primary care facilities.
		(b) Indicate number of participants with missing data for each variable of interest	Not Applicable	
		(c) *Cohort study*—Summarise follow-up time (eg, average and total amount)	Not Applicable	
Outcome data	15*	*Cohort study*—Report numbers of outcome events or summary measures over time	Not Applicable	
		Case-control study—Report numbers in each exposure category, or summary measures of exposure	Not Applicable	
		Cross-sectional study—Report numbers of outcome events or summary measures	Page 9-10, Line 250-260	Of the 1165 respondents, 66.70% enrolled in NCMS, and 23.00% and 10.30% enrolled in UEBMI and

For peer review only - http://bmjopen.bmj.com/site/about/guidelines.xhtml

				URBMI, respectively. The average expenditures per inpatient stay were 10,366 Chinese Yuan (=1,567 US dollars), and there was a small difference (about 500 Yuan, P=0.643) in inpatient expenditure between the NCMS and URBMI & UEBMI subsample. 66.78% of respondents who used inpatient care received reimbursement from social health insurance, with 60.49% for NCMS enrollees and 79.38% for URBMI & UEBMI enrollees. Among the respondents who received reimbursement, the average amount and ratio of the reimbursement received were 5,506 Yuan (=832 US dollars) and 46.77%. The average amount and ratio of the reimbursement received for NCMS enrollees were much smaller than those for URBMI & UEBMI enrollees.
Main results	16	(*a*) Give unadjusted estimates and, if applicable, confounder-adjusted estimates and their precision (eg, 95% confidence interval). Make clear which confounders were adjusted for and why they were included	Not Applicable	
		(*b*) Report category boundaries when continuous variables were categorized	Not Applicable	
		(*c*) If relevant, consider translating estimates of relative risk into absolute risk for a meaningful time period	Not Applicable	

Continued on next page

For peer review only - http://bmjopen.bmj.com/site/about/guidelines.xhtml

Other analyses	17	Report other analyses done—eg analyses of subgroups and interactions, and sensitivity analyses	Page 9, Line 238-240	All the analyses were conducted for the total sample, rural social insurance sample (the NCMS subsample), and urban social insurance sample (the URBMI & UEBMI subsample), respectively.
**Discussion**				
Key results	18	Summarise key results with reference to study objectives	Page 16-17, Line 389-426	Our main findings showed inequity in insurance benefits among migrants by migration scope…..Therefore, the inverse subsidies of health insurance funds from the less-developed out-migration regions to the highly-developed in-migration regions have become an ongoing challenge and policy dilemma faced by countries like China that has experienced mass domestic migration. Strategies that control health expenditures inflation and allow the central government to redistribute welfare fund to less-developed regions may be warranted to address the financial difficulties faced by out-migration regions
Limitations	19	Discuss limitations of the study, taking into account sources of potential bias or imprecision. Discuss both direction and magnitude of any potential bias	Page 18, Line 441-453	This study contained some limitations. First, the outcomes related to reimbursement were self-reported, leading to measurement

For peer review only - http://bmjopen.bmj.com/site/about/guidelines.xhtml

For peer review only

				bias. Future research will use health insurance claims data to minimize this bias. Second, health status may influence health care utilization and whether a person chooses to migrate. Studies have detected the healthy migrant effect for internal migrants in China, showing that healthier people were more likely to migrate and move farther away from home39. Unfortunately, there was no measurement of the health status of migrants in this dataset. Third, we could not accurately distinguish the administrative site of social health insurance, which may affect the insurance benefit for internal migrants. To reduce this bias, we conducted the analysis separately on two subsamples of NCMS enrollees and UEBMI & URBMI enrollees. Lastly, the 2014 China National Internal Migrant Dynamic Monitoring Survey is a dataset eight years ago, which may limit its implications for current issues.
Interpretation	20	Give a cautious overall interpretation of results considering objectives, limitations, multiplicity of analyses, results from similar studies, and other relevant evidence	Page 18-19, Line 456-473	This study has important policy implications not only for China but also for other developing countries that experience rapid urbanization and internal migration. In some

For peer review only

				developing countries…… Efforts should also be made to control inflation of health care expenditures and prevent inverse government subsidies from out-migration regions to in-migration regions.
Generalisability	21	Discuss the generalisability (external validity) of the study results	Not Applicable	
**Other information**				
Funding	22	Give the source of funding and the role of the funders for the present study and, if applicable, for the original study on which the present article is based	Page 20, Line 482-485	This work was supported by the National Natural Science Foundation of China (No. 71874034) and the National Key R&D Program of China (No. 2018YFC1312600 and 2018YFC1312604).The funders had no role in study design, data collection and analysis, preparation of the paper, or the decision to publish.

*Give information separately for cases and controls in case-control studies and, if applicable, for exposed and unexposed groups in cohort and cross-sectional studies.

Note: An Explanation and Elaboration article discusses each checklist item and gives methodological background and published examples of transparent reporting. The STROBE checklist is best used in conjunction with this article (freely available on the Web sites of PLoS Medicine at http://www.plosmedicine.org/, Annals of Internal Medicine at http://www.annals.org/, and Epidemiology at http://www.epidem.com/). Information on the STROBE Initiative is available at www.strobe-statement.org.

For peer review only - http://bmjopen.bmj.com/site/about/guidelines.xhtml
